# Comparative analysis of full-length mitochondrial genomes of five *Skeletonema* species reveals conserved genome organization and recent speciation

**DOI:** 10.1186/s12864-021-07999-z

**Published:** 2021-10-15

**Authors:** Shuya Liu, Yichao Wang, Qing Xu, Mengjia Zhang, Nansheng Chen

**Affiliations:** 1 Key Laboratory of Marine Ecology and Environmental Sciences, Institute of Oceanology, Chinese Academy of Sciences, 266071 Qingdao, China; 2 Laboratory of Marine Ecology and Environmental Science, Qingdao National Laboratory for Marine Science and Technology, Qingdao, 266237 China; 3 Center for Ocean Mega-Science, Chinese Academy of Sciences, Qingdao, 266071 China; 4 University of Chinese Academy of Sciences, Beijing, 10039 China; 5 College of Life Science and Technology, Huazhong Agricultural University, Wuhan, 430070 China; 6 Department of Molecular Biology and Biochemistry, Simon Fraser University, 8888 University Drive, Burnaby, British Columbia V5A 1S6 Canada

**Keywords:** Harmful algal blooms, *Skeletonema* species, Mitochondrial genome, Comparative genomics, Molecular marker, Divergence time

## Abstract

**Background:**

*Skeletonema* species are prominent primary producers, some of which can also cause massive harmful algal blooms (HABs) in coastal waters under specific environmental conditions. Nevertheless, genomic information of *Skeletonema* species is currently limited, hindering advanced research on their role as primary producers and as HAB species. Mitochondrial genome (mtDNA) has been extensively used as “super barcode” in the phylogenetic analyses and comparative genomic analyses. However, of the 21 accepted *Skeletonema* species, full-length mtDNAs are currently available only for a single species, *S. marinoi*.

**Results:**

In this study, we constructed full-length mtDNAs for six strains of five *Skeletonema* species, including *S. marinoi*, *S. tropicum*, *S. grevillei*, *S*. *pseudocostatum* and *S. costatum* (with two strains), which were isolated from coastal waters in China. The mtDNAs of all of these *Skeletonema* species were compact with short intergenic regions, no introns, and no repeat regions. Comparative analyses of these *Skeletonema* mtDNAs revealed high conservation, with a few discrete regions of high variations, some of which could be used as molecular markers for distinguishing *Skeletonema* species and for tracking the biogeographic distribution of these species with high resolution and specificity. We estimated divergence times among these *Skeletonema* species using 34 mtDNAs genes with fossil data as calibration point in PAML, which revealed that the *Skeletonema* species formed the independent clade diverging from *Thalassiosira* species approximately 48.30 Mya.

**Conclusions:**

The availability of mtDNAs of five *Skeletonema* species provided valuable reference sequences for further evolutionary studies including speciation time estimation and comparative genomic analysis among diatom species. Divergent regions could be used as molecular markers for tracking different *Skeletonema* species in the fields of coastal regions.

**Supplementary Information:**

The online version contains supplementary material available at 10.1186/s12864-021-07999-z.

## Background

*Skeletonema* is a species-rich genus of centric planktonic diatoms that belongs to the order Thalassiosirales. However, before the recognition/delimitation of eight *Skeletonema* species based on morphological features using light microscope, scanning and transmission electron microscope, and some molecular features, *Skeletonema* strains were usually regarded as a single species, *S. costatum* [1]. Since then, 21 *Skeletonema* species have been identified and taxonomically accepted [2]. Most *Skeletonema* species are cosmopolitan distributions, and are important primary producers especially in estuarine and marine waters [3]. For example, *S. marinoi* is particularly dominant in temperate coastal regions, including the Bohai Sea and the Yellow Sea [4, 5], West Sea of Korea [6], Gullmar Fjord, Sweden [7], Baltic Sea [8], and Adriatic Sea [9]. *S. grevillei* has wide geographical distributions but low abundance [3], even though it has been recorded in Adriatic Sea [10], Arabian Sea [3], Mediterranean [9], Xiamen Harbour in China [3, 11] and Hong Kong Bay in China [12]. *S. tropicum* were found at tropical locations including Mediterranean Sea [3], the East China Sea [3], the South China Sea [6], the Jiaozhou Bay of the Yellow Sea [13], and the Lagoa dos Patos in Brazil during summer and autumn [3]. *S. pseudocostatum* is often observed with unicellular or short chained forms, and is also associated with long threads, has been recorded in Australian, South African, Brazilian and Chinese waters as well as the Mediterranean, Baltic Seas and Sontecomapan lagoon, Mexico [3, 14, 15]. *S. pseudocostatum* has more restricted geographical ranges because the species was not found along American coasts [3]. *S. potamos* grows in freshwaters and brackish waters with salinities 2–34 [16], such as the River Danube, Hungary [17] and Patos Lagoon, Brazil [18].

Because of their worldwide distribution and critical importance in ecology and aquaculture, many *Skeletonema* species have been extensively studied [19, 20]. For example, research found that *Skeletonema* species are important regulators of global climate by playing an important role in the biochemical cycling of carbon and oxygen and various nutrients [21]. Under certain circumstances, many *Skeletonema* species can induce harmful algal blooms (HABs) [1], some of which can cause severe economic losses among aquaculture, fisheries and tourism industries, as well as ecological structure throughout the world [22]. *Skeletonema* HABs, which are usually incorrectly described as *S. costatum* HABs, are frequent with negative consequences [23–25]. For example, the most dominant *Skeletonema* species in the Jiaozhou Bay, China was generally described as *S. costatum* [5]. Most *Skeletonema* strains isolated from the Jiaozhaou Bay were identified as *S. marinoi* by molecular markers [26]. *Skeletonema* HABs species could cause hypoxia or anoxia after reaching dense concentrations leading to indiscriminate mortalities in marine life [27]. *Skeletonema* HABs can also cause severe loss in the aquaculture. For example, the *Skeletonema* HABs utilize nutrients necessary for the growth of the red algae *Porphyra* in winter in Japanese aquaculture [20]. *Skeletonema* HABs have been described to occur worldwide. For example, in one of the best studied ocean region the Jiaozhou Bay, *Skeletonema* species was found to be the most dominant phytoplankton, and *Skeletonema* HABs were found to occur with the highest frequency over the past 84 years (1936–2019) [5]. Similarly, *Skeletonema* was one of the most frequently occurring diatom genus along Guangdong coast of the South China Sea during 1980–2016 [25].

In addition to their important role in ecology, some *Skeletonema* species are commonly used in aquaculture for feeding bivalves and crustaceans due to their good nutrient composition and absence of toxins [28]. As *Skeletonema* species are easily cultured, they are also often used as model systems for studying phytoplankton species [1]. For example, Johansson et al. (2019) [29] proposed *S. marinoi* as a genetic model for various attributes of marine diatoms, including grazer effects [30], sexual reproduction [31], programmed cell death [32], herbicide effects [33] and temperature acclimation [34]. *S. marinoi* has also been used in micro-evolutionary studies of temporal changes in diatom ecology and genetics, because it has a benthic resting stage that can survive in sediments for more than 100 years [35, 36].

Despite their ecological importance and aquacultural values, the biodiversity of *Skeletonema* species have not been well characterized due primarily to their high morphological similarities, especially for closely related species pairs such as *S. marinoi* and *S. dohrnii* [37], and *S. menzelii* and *S. tropicum* [3]. The application of molecular analysis using molecular markers, including full-length 18S rDNA and partial-length 28S rDNA (D1-D3), along with comparative analysis of morphological features, enabled the identification of eight *Skeletonema* species including *S. menzelii*, *S. pseudocostatum*, *S. subsalsum*, *S. tropicum*, *S. dohrnii*, *S. grethae*, *S. japonicum* and *S. marinoi* [1, 38, 39]. Nevertheless, common molecular markers such as partial 18S rDNA (V4 region) in environmental metabarcoding analyses using the second generation sequencing technology cannot adequately distinguish *Skeletonema* species [26]. *cox1* was shown to be the most useful marker compared with 18S rDNA and 28S rDNA D1-D3 in *Skeletonema* species identification [40], while the insufficient sequences of full length *cox1* gene in the NCBI database limited their application.

Because of their small sizes, conserved gene components, low level of recombination and maternal inheritance, mitochondrial genomes (mtDNAs) have been extensively used in the phylogenetic analysis, population genetics and biogeographic distribution [41]. mtDNAs have been used as “super barcode” for comparative genomics analysis [42]. Furthermore, mtDNAs usually show relatively higher evolutionary rates than nuclear genomes, which make them an appropriate platform for developing molecular markers with high resolution [43, 44]. By now, mtDNA of only a single *Skeletonema* species, *S. marinoi*, has been constructed [45].

In this study, we constructed mtDNAs of six *Skeletonema* strains (from five  *Skeletonema* species), including *S. marinoi*, *S. tropicum*, *S. grevillei*, *S*. *pseudocostatum* and *S. costatum* (two strains), which were isolated from coastal waters in China. Comparative analysis of these mtDNAs revealed high gene and synteny conservation, as well as segments of high divergence, which could be used to develop molecular markers for distinguishing *Skeletonema* species. We also explored divergence times for *Skeletonema* species and other species in the class Mediophyceae based on their shared protein-coding genes (PCGs) of mtDNAs.

## Results

### Morphological and molecular identification of six *Skeletonema* strains

**Fig. 1:**
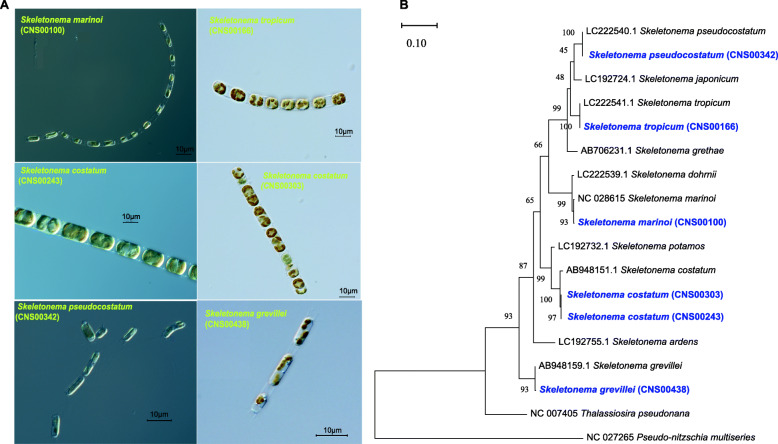
**A** Micrographs of six *Skeletonema* strains. **B** Phylogenetic tree of *Skeletonema* inferred from *cox1* genes. The six *Skeletonema* strains in blue were obtained in this study, and their *cox1* sequences have been uploaded to NCBI (MW438979-MW438984); the other sequences were downloaded from NCBI GenBank. Phylogenetic trees were generated using the Maximum Likelihood (ML) method with 1000 bootstrap replicates. Evolutionary model was Tamura-Nei model with gamma distribution (G = 0.187)

All candidate *Skeletonema* strains, which were readily identified by its cylindrical cells, usually formed long or short chains (Fig. 1A). *S. pseudocostatum* was observed with unicellular or two-three cells chains. Cell sizes of the candidate *Skeletonema* species varied substantially, which were not surprising because diatom cell size decreases as a result of the mechanism of cell division [31]. Thus, cell sizes alone could not be used as defining features for identifying *Skeletonema* species. Molecular information and more elaborate morphological characteristics are needed to facilitate species identification.

The *cox1* gene sequences of all six *Skeletonema* strains (Fig. 1B) were used in the initial annotation of the *Skeletonema* strains. The *cox1* gene sequence of the strain CNS00100 was identical to that of NC_028615.1 (percentage identity PID was 99.74%; Additional file 1), which was annotated as the species *S. marinoi*. Similarly, the strains CNS00166, CNS00243, CNS00303, CNS00342, and CNS00438 were annotated as *S. tropicum* (LC222541.1, PID = 100.00%), *S. costatum* (AB948151.1, PID = 99.20%), *S. costatum* (AB948151.1, PID = 99.20%), *S. pseudocostatum* (LC222540.1, PID = 100.00%) and *S. grevillei* (AB948159.1, PID = 99.90%; Additional file 1), respectively. The strains CNS00243 and CNS00303 were annotated to the same species, i.e. *S. costatum*.

Annotation using other molecular markers (18S rDNA, *rbcL* and 28S rDNA D1-D3) generally supported the *cox1*-based annotation of these six *Skeletonema* strains (Additional file 1; Additional file 2).

### Construction and analysis of *Skeletonema* mtDNAs

We constructed full-length mtDNAs for five *Skeletonema* species including *S. marinoi*, *S. pseudocostatum*, *S. grevillei*, *S. costatum* (with two strains), and *S. tropicum.* Notably, mtDNAs of *S. pseudocostatum*, *S. grevillei*, *S. costatum*, and *S. tropicum* were constructed in this project for the first time. Among the 36 reported mtDNAs in the Bacillariophyta (Additional file 3), 32 mtDNAs were full-length. The sizes of mtDNAs among the Bacillariophyta varied greatly (Additional file 4), ranging from 32,777 bp in *Melosira undulata* [46] to 103,605 bp in *Halamphora calidilacuna* [46]. The sizes of *Skeletonema* mtDNAs were relatively short, varying from 36,199 bp to 40,676 bp (Table 1). The AT contents were relatively similar (70.3–71.5%). All six *Skeletonema* mtDNAs contained 35 PCGs including *atp6, 8, 9; cob; cox1–3; nad1–7, 4 L, 9, 11; rpl2, 5, 6, 14, 16; rps 2, 3, 4, 7, 8, 10, 11, 12, 13, 14, 19*; and *tatA* and *tatC*), and two non-coding rRNA genes (*rns* and *rnl*; Table 1). Additionally, mtDNAs of *S. tropicum, S. grevillei, S. pseudocostatum* and *S. marinoi* contained 25 tRNAs, while that of *S. costatum* had 26 tRNAs and contain one more *trnM*. The start codon of *atp8* gene in *S. marinoi* was GTG, while the start codon of *atp8* was ATA in *S. tropicum* and *S. pseudocostatum*, and TTA in *S. costatum* and *S. grevillei*. No introns were found in any of these *Skeletonema* mtDNAs, although introns have been identified in many phytoplankton mtDNAs [46]. The intergenic regions of the mtDNAs of these five *Skeletonema* species were relatively small, with average sizes ranging from 58 bp in *S. tropicum* to 87 bp in *S. marinoi*, suggesting that the *Skeletonema* mtDNAs were all compact as other diatom mtDNAs [43].

**Table 1:** The genome features and gene content of 12 mtDNAs in the class Mediophyceae belonging to the Phylum Bacillariophyta. The strains indicated in blue were obtained in this study

Species	Strains	Habitat	Accession number	Size(bp)	A + T(%)	PCGs/rRNAs	rps2	rrn5	tRNA	Introns	Start codon of atp8	Reference
***Skeletonema marinoi***	CNS00100	Marine	MW438984	38,773	70.3	34/2	+	–	25	0	GTG	This study
*Skeletonema marinoi*	JK029	Marine	NC_028615	38,515	70.3	34/2	+	–	25	0	GTG	[45]
***Skeletonema tropicum***	CNS00166	Marine	MW438983	37,543	70.6	34/2	+	–	25	0	ATA	This study
***Skeletonema costatum***	CNS00243	Marine	MW438982	40,676	71.5	34/2	+	–	26	0	TTA	This study
***Skeletonema costatum***	CNS00303	Marine	MW438981	40,675	71.5	34/2	+	–	26	0	TTA	This study
***Skeletonema pseudocostatum***	CNS00342	Marine	MW438980	36,199	70.6	34/2	+	–	25	0	ATA	This study
***Skeletonema grevillei***	CNS00438	Marine	MW438979	36,336	70.8	34/2	+	–	25	0	TTA	This study
*Thalassiosira pseudonana*		Marine	NC_007405	43,827	69.9	34/2	+	–	25	1	TTA	[47]
*Thalassiosira profunda*	CNS00050	Marine	MW013551	40,470	69.0	34/2	+	–	25	0	ATT	[48]
*Odontella regia*	CNS00380	Marine	MW018491	37,617	73.4	34/2	–	+	24	0	ATG	[43]
*Lithodesmium undulatum*	CNS00316	Marine	MW023083	37,057	75.3	34/2	–	+	25	0	ATT	[43]
*Toxarium undulatum*	ECT3802	Marine	NC_037988	40,429	69.9	34/2	+	–	26	0	ATG	[49]

The core genes included 34 PCGs (*atp6*, *8*, *9*; *cob*; *cox1*, *2*, *3*; *nad1*–*7*, *4 L*, *9*, *11*; *rpl2*, *5*, *6*, *14*, *16*; *rps 3*, *4*, *7*, *8*, *10*, *11*, *12*, *13*, *14*, *19*; and *tatA*, *tatC*) and two rRNAs

### Phylogenetic analysis of *Skeletonema* mtDNAs

**Fig. 2:**
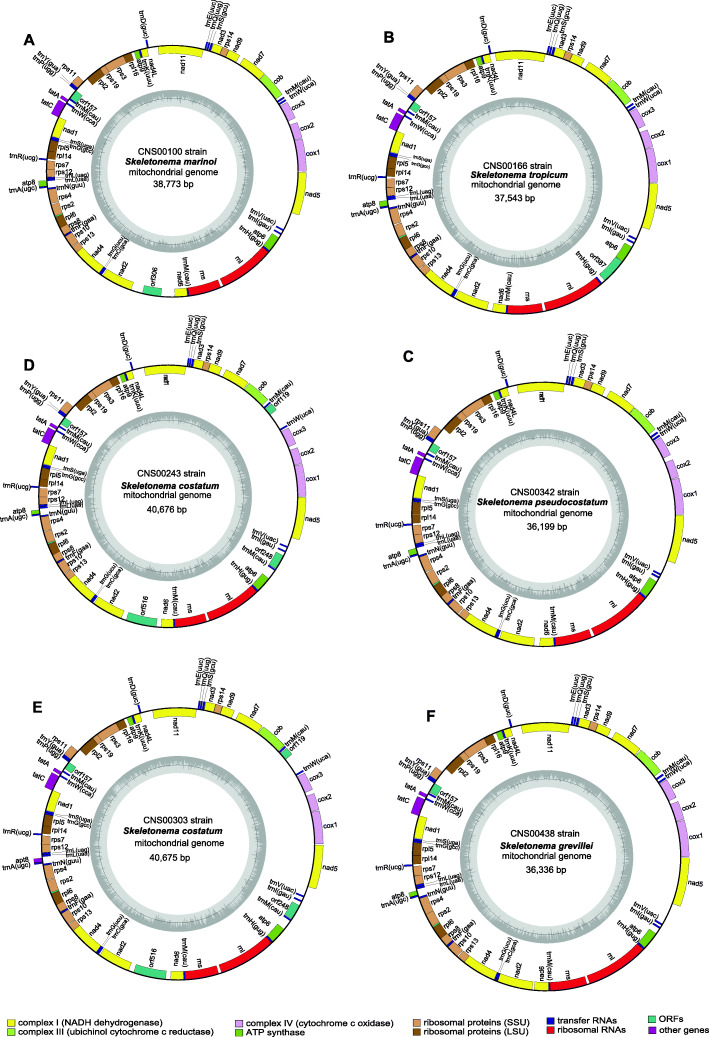
Circular maps of the complete mtDNAs of six *Skeletonema* species. The PCGs, rRNAs and tRNAs are labeled outside the circles. The assignment of genes is indicated by colors according to the different functional groups. The ring of bar graphs inside the circle shows the GC content in dark gray

**Fig. 3:**
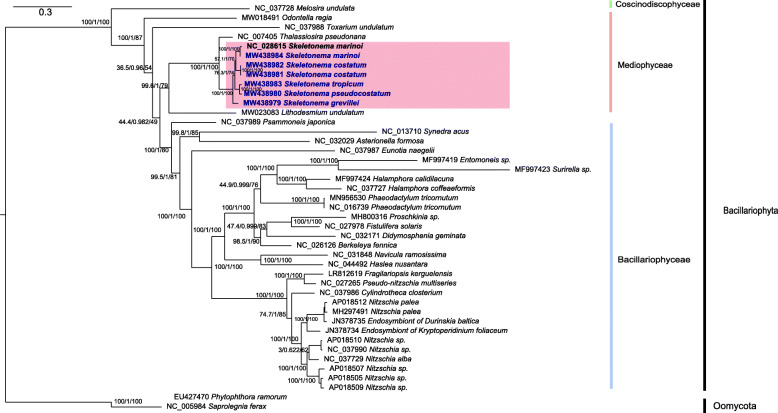
The phylogenetic tree based on concatenated amino acid sequences of 31 mitochondrial protein-coding genes (*atp6, 8, 9; cob; cox1–3; nad1–7, 4 L, 9, 11; rpl2, 5, 6, 14, 16; rps 3, 4, 8, 10, 11, 13, 14, 19*; and *tatC*) using Maximum likelihood (ML) methods. The branch values were SH-aLRT support (%) / aBayes support / ultrafast bootstrap support (%). The strains indicated in blue were obtained in this study. *Phytophthora ramorum* and *Saprolegnia ferax* were used as outgroups

Among the 36 Bacillariophyta species with reported mtDNAs, the five *Skeletonema* species belonged to the class Mediophyceae (Additional file 3; Table 1; Fig. 2), including *S. marinoi*, which is the only *Skeletonema* species whose full-length mtDNA has been constructed. To explore the evolutionary relationship between *Skeletonema* species and other diatom species, we constructed a phylogenetic tree using 31 PCGs that were shared by mtDNAs of Bacillariophyta and Oomycota, which were used as outgroup taxa (Fig. 3). All species in Bacillariophyta fell into three clades corresponding to three classes including Mediophyceae, Bacillariophyceae, and Coscinodiscophyceae (Fig. 3). In the class Mediophyceae, *Skeletonema* species clustered closely with *Thalassiosira pseudonana*, which was consistent with previous studies [17, 45]. Both genera belong to the same Order Thalassiosirales [2]. Within *Skeletonema*, the *S. marinoi* mtDNA constructed in this study (MW438984) clustered closely with that of the other *S. marinoi* strain (JK029 strain from Korean seawater, NC_028615) as expected, so were the mtDNAs of two *S. costatum* strains (CNS00243 and CNS00303). *S. tropicum* clustered closely with *S. pseudocostatum*, consistent with previous report [3, 24]. The mtDNA of *S. grevillei*, which was isolated from the South China Sea, was independent from other clades.

### Synteny conservation with divergent regions in mitochondrial genome

**Fig. 4:**
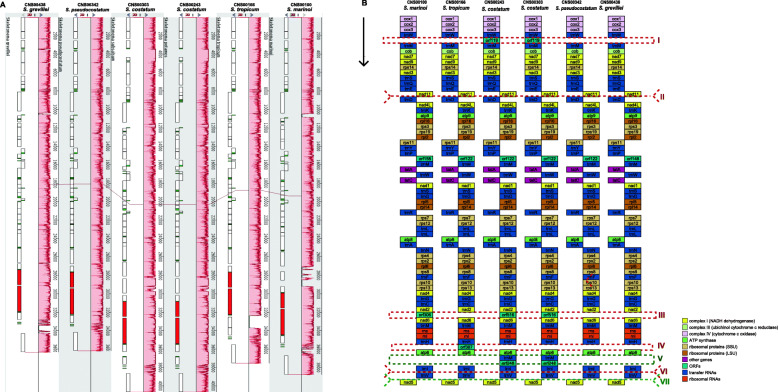
**A** Synteny relationships among six *Skeletonema* mtDNAs based on Mauve analysis. **B** mtDNA gene arrangements of six *Skeletonema* species. Blocks with the same color represent the same type of genes

The mtDNA sizes of these *Skeletonema* species showed some variations with the difference between the longest mtDNA (*S. costatum*, CNS00243 strain, 40,676 bp) and the shortest mtDNA (*S*. *pseudocostatum*, 36,199 bp) being 4477 bp. However, comparative analysis found that there was nearly perfect synteny conservation among *Skeletonema* species (Fig. 4A). This is expected because previous study found high synteny conservation between the mtDNAs of *S. marinoi* and *T. pseudonana* [43], which is diatom species of a different genus in the order Thalassiosirales. At the single gene level, the genome organization was also well conserved among the five *Skeletonema* species with only minor differences (Fig. 4B). The numbers of open reading frames (*orf*s) and *trnM* genes were different among these *Skeletonema* mtDNAs, which were primarily responsible for the different lengths of mtDNAs. *S. pseudocostatum* and *S. grevillei* each had a single *orf*, *S. marinoi* and *S. tropicum* each had two *orf*s, while *S. costatum* each had three *orf*s. *S. costatum* each had an additional *trnM*. Compared with *S*. *pseudocostatum*, mtDNA of *S. costatum* had three more *orfs* (*orf119*, *orf516* and *orf248*) and one more *trnM*, which in total accounted for 2730 bp. Another main reason for the big size difference between the two species was the length of intergenic regions. For example, the length of the combined intergenic regions in *S. costatum* (CNS00243 strain) was roughly 1745 bp longer than that of *S*. *pseudocostatum.*

**Fig. 5:**
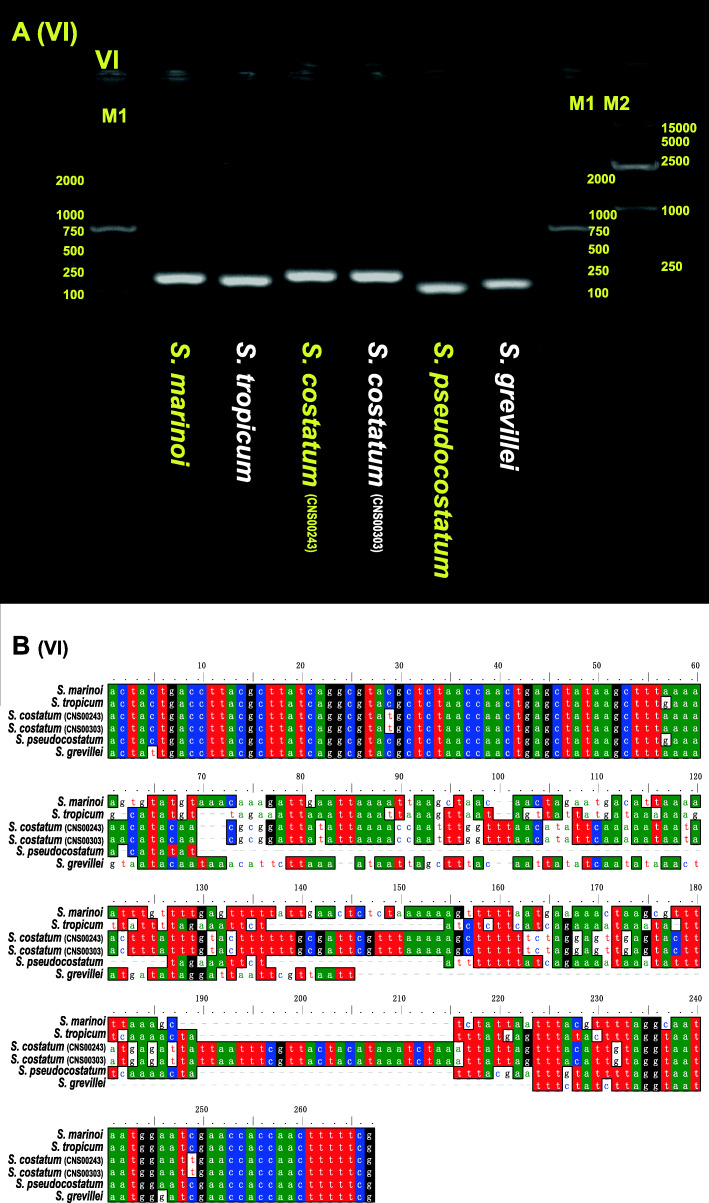
**A** Agarose gels image of PCR products for region VI among the six *Skeletonema* mtDNAs. The full-length gels are presented in Additional file 15. The brands sequences were *S. marinoi*, *S. tropicum*, *S. costatum* (CNS00243)*, S. costatum* (CNS00303), *S. pseudocostatum* and *S. grevillea*, respectively. The brands of Marker M1 and M2 were on the right. **B** DNA alignment information of region VI for the six *Skeletonema* species

Interestingly, despite the deep conservation of these mtDNAs, comparative sequence analysis of these mtDNAs also revealed seven discrete regions with enhanced divergence, which were indicated using I, II, III, IV, V, VI and VII (Fig. 4B). Within these regions, DNA sequences were essentially unalignable (Additional file 5 and Additional file 6). Region I was located between *trnW* and *trnM* (Additional file 5A). The *S. costatum* mtDNAs have one big insertion (*orf119*) compared with other species, which were also validated by designed PCR primers (I-F and I-R in Additional file 7; Additional file 6). Insertions in mtDNAs of *S. costatum* (*trnM* and *orf248*) were also found in region V (located between *atp6* and *trnI*; Additional file 5E and Additional file 6), which were validated by PCR primers (V-F and V-R in Additional file 7). In region III (located between *nad2* and *nad6*; Additional file 5C and Additional file 6), the mtDNAs of *S. marinoi* (*orf306*), and *S. costatum* (*orf516*) had big insertions, respectively. In the region IV, only *S. tropicum* mtDNA had an insertion (*orf387* between *trnH* and *atp6*) with 1786 bp length compared with other *Skeletonema* species with the lengths between 439 and 455 bp (Additional file 5D and Additional file 6). Divergent regions II, VI and VII, were located between *nad11* and *trnD* (Additional file 5B and Additional file 6); between *trnI* and *trnV* (Fig. 5A-B; Additional file 5F); between *trnV* and *nad5* (Additional file 5G), respectively. These divergent regions (Fig. 4B and Additional file 5) are generally located at the non-coding regions except the region V. The nature regarding the formation of these divergent regions remained unknown. However, these divergent regions may be used as potential molecular markers for distinguishing different *Skeletonema* species.

Among the seven regions, region VI could be used a molecular marker for distinguishing five *Skeletonema* species with both high resolution and high specificity. This region was different among five *Skeletonema* species, thus it could be used to distinguish different *Skeletonema* species (Fig. 5). To explore the specificity of the region VI as a molecular marker, primers (VI-F: ACTACTGACCTTACGCTTATC, VI-R: CGAAAAAGTTGGTGGTTCGATTCCA) were designed for PCR amplification of the region VI in 11 other phytoplankton species (i.e., *Thalassiosira pseudonana, Chaetoceros muelleri, Heterosigma akashiwo, Aureococcus anophagefferens, Chattonella marina, Amphidinium carterae, Alexandrium tamarense, Karenia mikimotoi, Prorocentrum donghaiense, Isochrysis galbana*, and *Phaeocystis globosa*). Agarose gel images of PCR products showed that only the PCR amplification was positive only for *Chaetoceros muelleri* with one bright band, whose length was much longer than that of *Skeletonema* species (Additional file 8), suggesting that the region VI can be used as a molecular marker to distinguish *Skeletonema s*pecies with high specificity.

### Divergence time estimation based on PCGs of mtDNAs

**Fig. 6:**
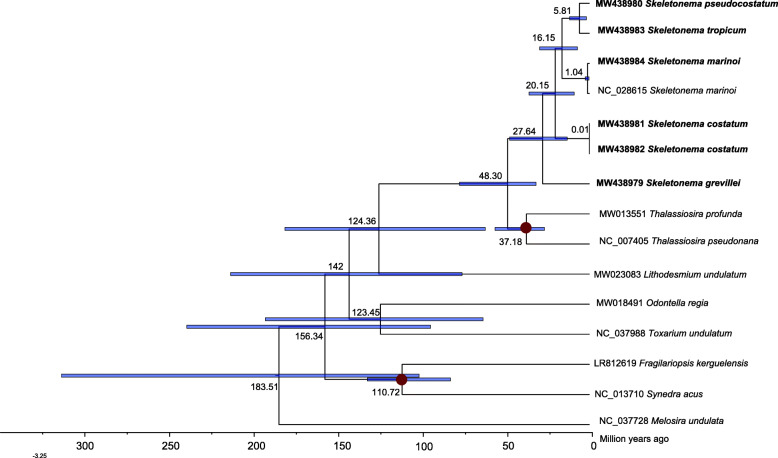
Time-calibrated phylogeny of 15 mtDNAs based on 34 PCGs in the class Mediophyceae and outgroup (*Melosira undulata*). We added two Bacillariophyceae species (*Synedra acus* and *Fragilariopsis kerguelensis*) to establish appropriate fossil calibration points. The red dots represent calibration points and the 95% highest posterior density interval for node ages are shown with translucent blue bars

The time-calibrated phylogeny of the Mediophyceae and other species was constructed using DNA sequences of 34 PCGs from 15 mtDNAs. The divergence times of Mediophyceae species were estimated using the MCMC tree (Fig. 6). The divergence between the class Mediophyceae and the class Coscinodiscophyceae was inferred to have occurred at 183.51 Ma ago (Mya; 95% HPD (highest posterior density): 100.65–312.13 Mya). Furthermore, the *Skeletonema* species formed a monophyletic clade at approximately 48.30 Mya (95% HPD 31.52–76.79 Mya) apart from *Thalassiosira* species. Among the *Skeletonema* species: *S. grevillei* diverged from other *Skeletonema* species approximately 27.64 Mya (95% HPD 13.10–47.11 Mya). The sister clade of *S. costatum* with two strains diverged from the clades including *S. marinoi*, *S. tropicum* and *S. pseudocostatum* approximately 20.15 Mya (95% HPD 8.94–35.6). *S. marinoi* diverged from the sister clade between *S. tropicum* and *S. pseudocostatum* approximately 16.15 Mya (95% HPD 7.08–29.41 Mya).

## Discussion

### Compact *Skeletonema* mtDNAs

The six *Skeletonema* mtDNAs constructed in this study had relatively small sizes (Additional file 4), with short intergenic regions, no introns, and no repeat regions. Introns have been found in many diatom mtDNA genes (Additional file 3), which greatly increased the length of mtDNA. For example, the largest mitochondrial genome ever discovered was *Halamphora calidilacuna* [46], reaching 103,605 bp. The *cox1* genes of *H. calidilacuna* spanned 31,716 bp, with only 1506 bp (4.7% of the total length of *cox1* gene) coding sequences. Some repeats were also found in some mtDNAs, which induced larger sizes of mtDNAs. For example, mtDNA of *Phaeodactylum tricornutum* contained a 35 kb-long repeat [50]. The intergenic regions also contribute significantly to the whole length of mtDNAs, and the larger size of intergenic regions is usually associated with larger size of mtDNAs. Thus another reason for the small size mtDNAs among *Skeletonema* species was relatively short intergenic regions compared to other diatom species (Additional file 4).

### Synteny conservation of *Skeletonema* mtDNAs

Our analysis found that *Skeletonema* mtDNAs contained similar numbers of genes containing 35 common PCGs, 2 rRNA genes and 25 tRNA genes (except in the *S. costatum*, which contained an additional *trnM* gene), same as the two mtDNAs of *Thalassiosira* (*T. pseudonana* and *T. profunda*) [47, 48]. The *Nitzschia* mtDNAs also include 35 unique PCGs and 2 rRNA genes, but these mtDNAs only have 24 tRNA genes compared that in *Thalassiosira* with and *Skeletonema* genus [46, 49, 51, 52]. Interesting, the mtDNAs in *Halamphora* genus show difference: *H. calidilacuna* mtDNA includes 34 PCGs and 26 tRNA genes; while *H. coffeaeformis* mtDNA includes 35 PGCs and 24 tRNA genes [46]. The mtDNAs of the two *Halamphora* species, which were quite different from that of the above three genera, include 3 rRNA genes and introns. While for the genetic structure, the mtDNAs belonging to the same genus showed highly synteny although the number of genes and genome sizes within the genus varied greatly (Additional file 9 and Fig. 4A). We also found slight variation among the *Thalassiosira* genus with two inversions (Additional file 9), showing the interspecific variability in the *Thalassiosira* genus. In brief, the mtDNAs of five *Skeletonema* species showed high conservation, which was in agreement with other species belonging to the same genus according to the previously reported mtDNAs.

### Phylogeny and evolution of *Skeletonema* species

Diatoms arose in the Triassic period, perhaps as early as 250 Mya according to the molecular clock estimate [53], and provided a main ecological role in carbon cycle about 100 Mya, when the CO_2_ level was five times higher than that nowadays. The timing coincided with the divergence of a second major lineage of diatoms, the bipolar and multipolar centrics, including Thalassiosirales clade [54]. Phylogenetic analysis of 31 core PCGs showed that *Skeletonema* was sister to *Thalassiosira*, both belonging to the order Thalassiosirales, consistent with the current classification [2, 55]. Our results (Fig. 6) show that *Skeletonema* species diverged from *Thalassiosira* species approximately 48.30 Mya (95% HPD: 31.52–76.79 Mya) during the Eocene, a period included a major burst of diversification affected by sea level, silica availability and interspecific competition with other planktonic groups [56]. This divergence time was estimated using 34 mtDNA genes, which was slightly earlier than that estimated using the two nuclear genes, seven plastid genes and two mitochondrial genes [55, 57], but was later than that estimated using two nuclear genes and two plastid genes [58], and using 18S rDNA [59].

Among the *Skeletonema* genus, the two HAB species *S. marinoi* and *S*. *pseudocostatum* diverged approximately 16.15 Mya (95% HPD 7.08–29.41 Mya), which was slight early than that results estimated previously using 18S rDNA [53]; and were later than that estimated using the two nuclear genes, seven plastid genes and two mitochondrial genes [55]. The divergence times estimated based on 34 PCGs of mtDNA among *Skeletonema* species in this study were generally consistent with previous results with minor fluctuations, which facilitating the understanding the evolution of *Skeletonema* species in the view of mitochondria with uniparental inheritance.

## Conclusion

In this study, we reported mtDNAs of four *Skeletonema* species for the first time, and re-sequenced the *S. marinoi* mtDNA of a strain isolated in coastal waters in China. Through comparative analysis of the mtDNA structure, base composition and gene order, we found relatively conserved genetic arrangement among the *Skeletonema* species. We also reported discrete regions with high divergence among the mtDNAs of five *Skeletonema* species, which could potentially provide high resolution for the phylogenetic studies among the genus. We further reconstructed a phylogenetic tree, which was used to estimate the divergence times among *Skeletonema* species. However, more taxa and more complete mtDNAs data are needed to verify the evolutionary relationships and primers application.

The mtDNAs of five *Skeletonema* species showed similar sizes, conserved genetic structure and high syntenic relationships among the genus. While the five *Skeletonema* species showed different ecological geographical distribution (Additional file 10). For example, *S. marinoi* was easily found in seawaters of higher latitude with lower temperature (Additional file 10A); While *S. tropicum* was recorded in seawaters of lower latitude with higher temperature (Additional file 10B). We hypothesize that the nuclear genomes of the *Skeletonema* species may possess substantial differences, which facilitate the adaptation of different *Skeletonema* species. To further explore the specific ecological distribution mechanism, whole genomes of *Skeletonema* species could help to provide new ideas.

## Methods

### Strain isolation and whole genome sequencing

*Skeletonema* strains studied in this project were isolated from water samples collected during six expeditions in Chinese coastal waters (Additional file 11), using methods as described previously [43]. The seawater of culture was collected from Jiaozhou Bay, filtered through a 0.22 μm mixed-fiber membrane and sterilized at 121 °C for 30 min. The *Skeletonema* strains were maintained in L1 medium [60]. The culture salinity was 28–30 ‰. The culture temperature was 19 ± 1 °C, and the irradiance was from 72 μmol photons /(m^2^·s) with a 12:12 h light-dark cycle. The strains were maintained in our lab for 3 months to 13 months before sequencing.

The morphological features of the *Skeletonema* strains were observed using ZEISS Axio Imager 2 (ZEISS, Germany) at the 600× magnification. We selected reprehensive strains based on the preliminary morphological observation for Illumina sequencing. For DNA extraction, healthy cells at the exponential growth phase were harvested via centrifugation at 8000 rpm for 5 min. Total DNAs were extracted using DNAsecure Plant Kit (Tiangen Biotech, Beijing, China) using the manufacturers’ instructions. DNA samples were quantified using Qubit® DNA Assay Kit in Qubit® 3.0 Flurometer (Invitrogen, USA), 0.2 μg genomic DNA of each sample was fragmented by sonication (Covaris S220, Covaris, USA) to the length of 350 bp. DNA fragments were harvested, end polished, A-tailed, and ligated with adapters for Illumina sequencing, followed by PCR amplification. The PCR products were purified using AMPure XP system (Beckman Coulter, Beverly, USA). Qualified libraries were sequenced using NovaSeq PE150 (Illumina, San Diego, CA, USA) at Novogene (Beijing, China).

### Genomic assessment, assembly and annotation

Clean data was obtained after trimming using Trimmomatic-0.39 [61]. From the K-mer analysis using Jellyfish [62] and GenomeScope [63], estimated nuclear genome sizes of six *Skeletonema* strains ranged from 51.72 M (*S. grevillei*) to 119.42 M (*S. costatum*, CNS00303 strain) (Additional file 11 and Additional file 12). The heterozygosity ranged from 0.11 to 2.90%. The repetitive rate ranged from 0.83 to 2.54%. Bacterial contamination was negligible (< 0.3%) in the DNA samples of *S. marinoi*, *S. tropicum*, *S. costatum* (CNS00243 strain) and *S. grevillei* strains, while the bacterial contaminations were relatively high in DNA samples of *S.* *pseudocostatum* (20.64%) and *S. costatum* (CNS00303 strain, 18.58%) strains. Common molecular markers, including full-length of 18S rDNA, *cox1* and *rbcL*, 28S rDNA D1-D3 and complete mtDNAs were assembled using the GetOrganelle [64]. All of the assembled results were validated using BWA [65], SAMtools [66] and IGV [67].

MtDNAs were annotated using MFannot (https://github.com/BFL-lab/Mfannot), Annotation results were validated and corrected by the NCBI’s ORF Finder (https://www.ncbi.nlm.nih.gov/orffinder/) with the genetic code of 4 Mold, Protozoan, and Coelenterate Mitochondrial; Mycoplasma/Spiroplasma. For accurate comparison, we have also inspected and re-annotated mtDNAs downloaded from the NCBI (Additional file 3).

### Phylogenetic analysis and divergence time estimations

The Phylogenetic trees of *cox1* (Fig. 1), full-length 18S rDNA, *rbcL* and 28S rDNA D1-D3 (Additional file 1; Additional file 2) were generated using the Maximum Likelihood (ML) method with 1000 bootstrap replicates in MEGAX [68]. Appropriate evolutionary models were selected using Model Selection.

The phylogenetic tree of mitochondrial PCGs were constructed by extracting 31 PCGs, including *atp6, 8, 9; cob; cox1, 2, 3; nad1–7, 4 L, 9, 11; rpl2, 5, 6, 14, 16; rps3, 4, 8, 10, 11, 13, 14, 19* and *tatC*, from the published Bacillariophyta mtDNAs and six *Skeletonema* mtDNAs in this study (Additional file 3) [43, 48]. The 31 genes amino acid sequences were individually aligned using MAFFT [69], trimmed using trimAl 1.2rev59 [70], and concatenated using Phyutility [71]. The evolutionary models were obtained using ModelFinder [72]. The phylogenetic tree was constructed using IQ-TREE [73] with SH-aLRT support (%) / aBayes support / ultrafast bootstrap support (%). *Phytophthora ramorum* and *Saprolegnia ferax* in Oomycota were used as outgroups (Fig. 3). FigTree 1.4.3 (http://tree.bio.ed.ac.uk/software/figtree/) was used to display the phylogenetic tree.

Phylogenetic analysis and molecular dating were analyzed using 34 mitochondrial PCGs DNA sequences shared by Mediophyceae species and outgroups *Melosira undulata*. The 34 PCGs included *atp6*, *8*, *9*; *cob*; *cox1*–*3*; *nad1*–*7*, *4 L*, *9*, *11*; *rpl2*, *5*, *6*, *14*, *16*; *rps 3*, *4*, *7*, *8*, *10*, *11*, *12*, *13*, *14*, *19*; and *tatA* and *tatC*, which were shared among the 15 species. These PCGs were aligned (MAFFT with Codon mode) and concatenated (Concatenate Sequence). The best-fit evolutionary model and partitioning scheme were selected by PartitionFinder2, which were shown in the Additional file 13. The phylogenetic trees (MrBayes) was constructed using the software PhyloSuite v1.2.2 [74]. Molecular dating was performed using the PAML package [75] in there three steps: (1) Rough estimation of substitution rate was analyzed using baseml; (2) Estimation of gradient and hessian of branch length; (3) Estimation of divergence times with the approximate likelihood method using the mcmctree. Two calibration points used in this analysis were shown in the Additional file 14. We added two Bacillariophyceae species (*Synedra acus* and *Fragilariopsis kerguelensis*) to establish appropriate fossil calibration points. The calibration point of *Synedra* and *Fragilaria* was based on fossil record [57]. The divergence times with fossil-calibrated deriving from ribosomal RNA and organelle genes, i.e. *T. profunda* and *T. pseudonana* [53, 58, 76]. The phylogenetic tree was displayed in the FigTree and visualized with 95% HPD  interval for each node.

### Synteny analysis and molecular marker development of six *Skeletonema* mtDNAs

Synteny analysis of six *Skeletonema* mtDNAs sequences was carried out with the program Mauve v2.3.1 [77]. The region with great sequence differences were aligned using MUSCLE algorithm in the MEGAX. Six primers (I-F/R to VI-F/R in Additional file 7) for PCR amplification of molecular markers (region I to VI in Fig. 4B) were developed using the Primer Premier 5.0 [78]. PCRs were performed in a final volume of 50 μL containing 2 μL diluted template DNA (about 50 ng), 1 μL forward primer, 1 μL reverse primer, 25 μL mix (Tiangen, China) and 21 μL ddH_2_O. The reaction was denatured at 94 °C for 4 min. Then, reactions were run for 32 cycles at 94 °C for 1 min, 52 °C for 1 min 50s, and 72 °C for 2 min and a final extension at 72 °C for 10 min. These PCR products were run on 1% agarose gels for checking amplicon lengths.

## Supplementary Information

**Additional file 1. **The alignment results with highest PID for common molecular markers of six *Skeletonema* strains. PID means percentage identity.

**Additional file 2. **The phylogenetic analysis of *Skeletonema* species and outgroups using 18S rDNA (**A**), the D1-D3 region of 28S rDNA gene (**B**) and *rbcL* (**C**). Phylogenetic trees were generated using the Maximum Likelihood (ML) method with 1000 bootstrap replicates. Evolutionary models for 18S rDNA, the D1-D3 region of 28S rDNA gene, and *rbcL* were Tamura 3-parameter model with gamma distribution (G = 0.617), Kimura 2-parameter model with gamma distribution (G = 0.297), and Tamura 3-parameter with gamma distribution (G = 0.689).

**Additional file 3.** Genome features of 36 mtDNAs from the Phylum Bacillariophyta.

**Additional file 4.** The intergenic region sizes and mtDNAs sizes of completed mtDNAs in the Bacillariophyta.

**Additional file 5. **The alignment details of seven regions with great difference of six *Skeletonema* mtDNAs.

**Additional file 6. **The agarose gels image of PCR products of seven regions among the six *Skeletonema* mtDNAs. The full-length gels are presented in Additional file 15. For each region (I-VI), the brands sequences were *S. marinoi*, *S. tropicum*, *S. costatum* (CNS00243), *S. costatum* (CNS00303)*, S. pseudocostatum* and *S. grevillei*, respectively. The brands of Marker M1 and M2 were on the right.

**Additional file 7. **The primes for the region I to region VI among the *Skeletonema* genus. F in the primer names meant forward primer; R in the primer names meant reverse primer. I to VI in the primer names were corresponding to region I to region VI.

**Additional file 8. **The agarose gels image of PCR products for *cox1* and region VI among *Skeletonema* species and other eleven species (*Thalassiosira pseudonana, Chaetoceros muelleri, Heterosigma akashiwo, Aureococcus anophagefferens, Chattonella marina, Amphidinium carterae, Alexandrium tamarense, Karenia mikimotoi, Prorocentrum donghaiense, Isochrysis galbana* and *Phaeocystis globosa*). The primers of *cox1* gene were, F: GGAACTTTATATTTAATYTTTGGWGC, R: AATACCAGAATTAGCAAGAACAAC [40]. The full-length gels are presented in Additional file 16.

**Additional file 9. **Synteny relationships among two *Thalassiosira* mtDNAs (**A**); eight *Nitzschia* mtDNAs (**B**); two *Halamphora* mtDNAs (**C**) based Mauve analysis.

**Additional file 10. **The distributions of five *Skelentonema* species from GBIF (https://www.gbif.org/).

**Additional file 11. **The sample collection and genomic assessment information of six *Skeletonema* strains.

**Additional file 12. **The genomic assessment of six *Skeletonema* strains.

**Additional file 13.** The best-fit evolutionary model and partitioning scheme for phylogenetic analysis and molecular dating.

**Additional file 14.** Calibration points used in the divergence time analysis by PAML.

**Additional file 15.** The full-length gels used in Fig. 5 and Additional file 7.

**Additional file 16.** The full-length gels used in Additional file 9.

## Data Availability

The sequencing results (raw data) have been submitted to NCBI and the BioProject number is PRJN695365 (https://www.ncbi.nlm.nih.gov/sra/?term=PRJNA695365). The mtDNA sequences and annotation results of six *Skeletonema* species have been submitted to NCBI under specific accession numbers (MW438979-MW438984). The *Skeletonema* species are provided by the Key Laboratory of Marine Ecology and Environmental Science from the Institute of Oceanology, Chinese Academy of Sciences.

## Abbreviations

mtDNA: Mitochondrial genome
HAB: Harmful algal bloom
PCG: Protein-coding gene
HPD: Highest posterior density
*orf*: Open reading frame
