# Supplementary material for: Comparative analysis of full-length mitochondrial genomes of five Skeletonema species reveals conserved genome organization and recent speciation
Source: BMC Genomics. 2021 Oct 15;22:746. doi: 10.1186/s12864-021-07999-z (PMC8520197; doi:10.1186/s12864-021-07999-z)
Supplement: Supplementary file 1 — Additional file 1. The alignment results with highest PID for common molecular markers of six Skeletonema strains. PID means percentage identity. [file 12864_2021_7999_MOESM1_ESM.docx]

**Additional file 1:** The alignment results with highest PID for common molecular markers of six *Skeletonema* strains. PID means percentage identity. (Docx 18 kb)

|  | **18 SrDNA** | | | **28 SrDNA** | | | ***cox1*** | | | ***rbcL*** | | |
| --- | --- | --- | --- | --- | --- | --- | --- | --- | --- | --- | --- | --- |
|  | accession ID | PID | species | accession ID | PID | species | accession ID | PID | species | accession ID | PID | species |
| **CNS00100** | HM236346.1 | 99.83% | *S. marinoi* | AJ633529.1/DQ396510.1 | 99.62% | *S.marinoi/S. dohrnii* | NC_028615.1 | 99.74% | *S. marinoi* | KM594531.1 | 99.78% | *S. costatum* |
| **CNS00166** | KU363218.1 | 100.00% | *S. tropicum* | AJ633516.1 | 100% | *S. tropicum* | LC222541.1 | 100% | *S. tropicum* | DQ514818.1 | 99.24% | *S. grethae* |
| **CNS00243** | AB948141.1 | 99.89% | *S. costatum* | DQ396490.1 | 99.49% | *S. costatum* | AB948151.1 | 99.20% | *S. costatum* | DQ514820.1 | 99.73% | *S. subsalsum* |
| **CNS00303** | AB948141.1 | 100.00% | *S. costatum* | DQ396490.1 | 99.49% | *S. costatum* | AB948151.1 | 99.20% | *S. costatum* | DQ514820.1 | 99.73% | *S. subsalsum* |
| **CNS00342** | AY684958.1 | 99.89% | *S. pseudocostatum* | AJ633514.1 | 100% | *S. pseudocostatum* | LC222540.1 | 100% | *S. pseudocostatum* | MK372941.1 | 100% | *S. pseudocostatum* |
| **CNS00438** | AB948143.1 | 100.00% | *S. grevillei* | DQ396496.1 | 99.75% | *S. grevillei* | AB948159.1 | 99.90% | *S. grevillei* | DQ514822.1 | 98.24% | *S. japonicum* |
