# Supplementary figures and images for: Comparative analysis of full-length mitochondrial genomes of five Skeletonema species reveals conserved genome organization and recent speciation
Source: BMC Genomics. 2021 Oct 15;22:746. doi: 10.1186/s12864-021-07999-z (PMC8520197; doi:10.1186/s12864-021-07999-z)

**A** 18S rDNA

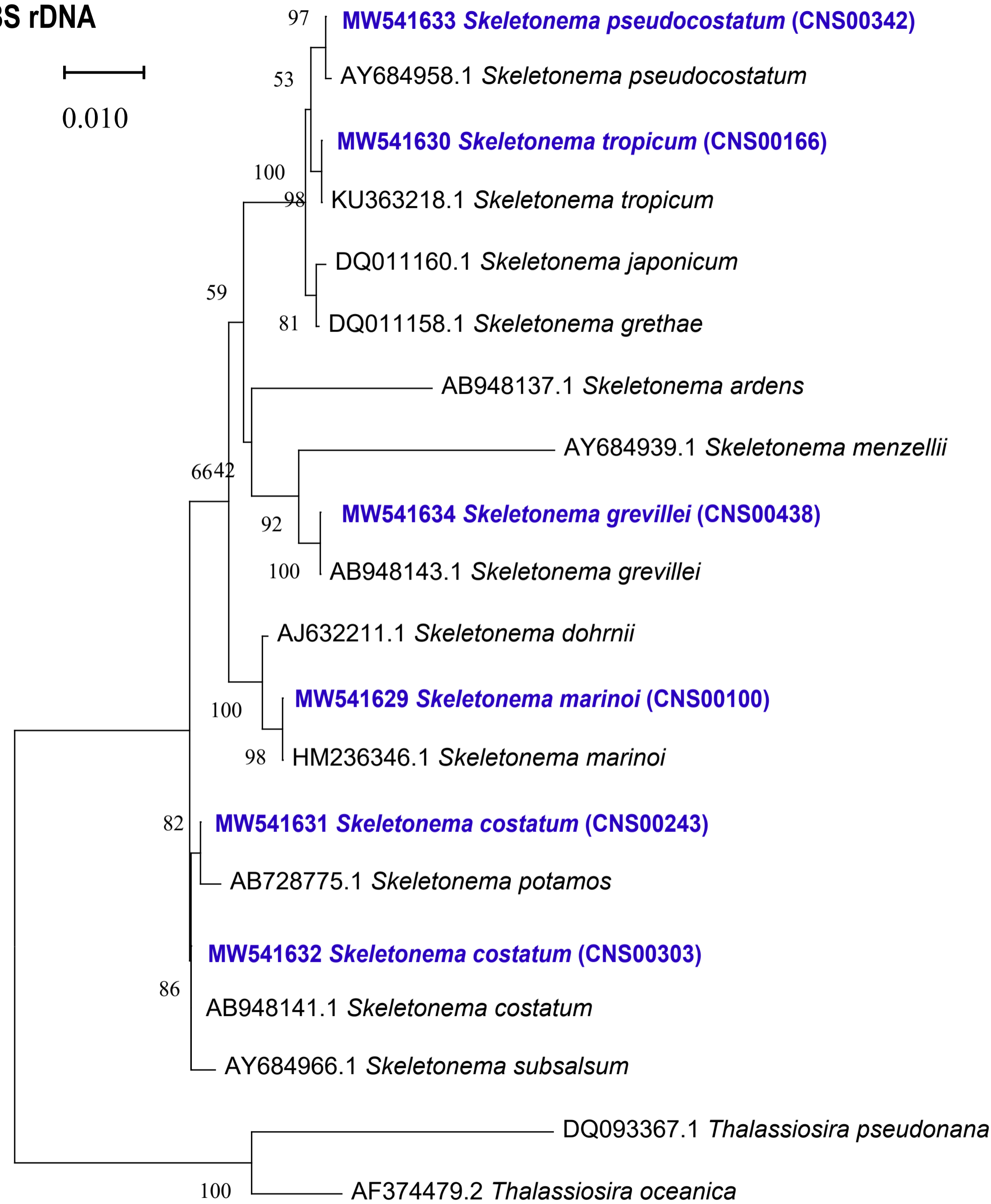

**B** 28S rDNA (D1-D3)

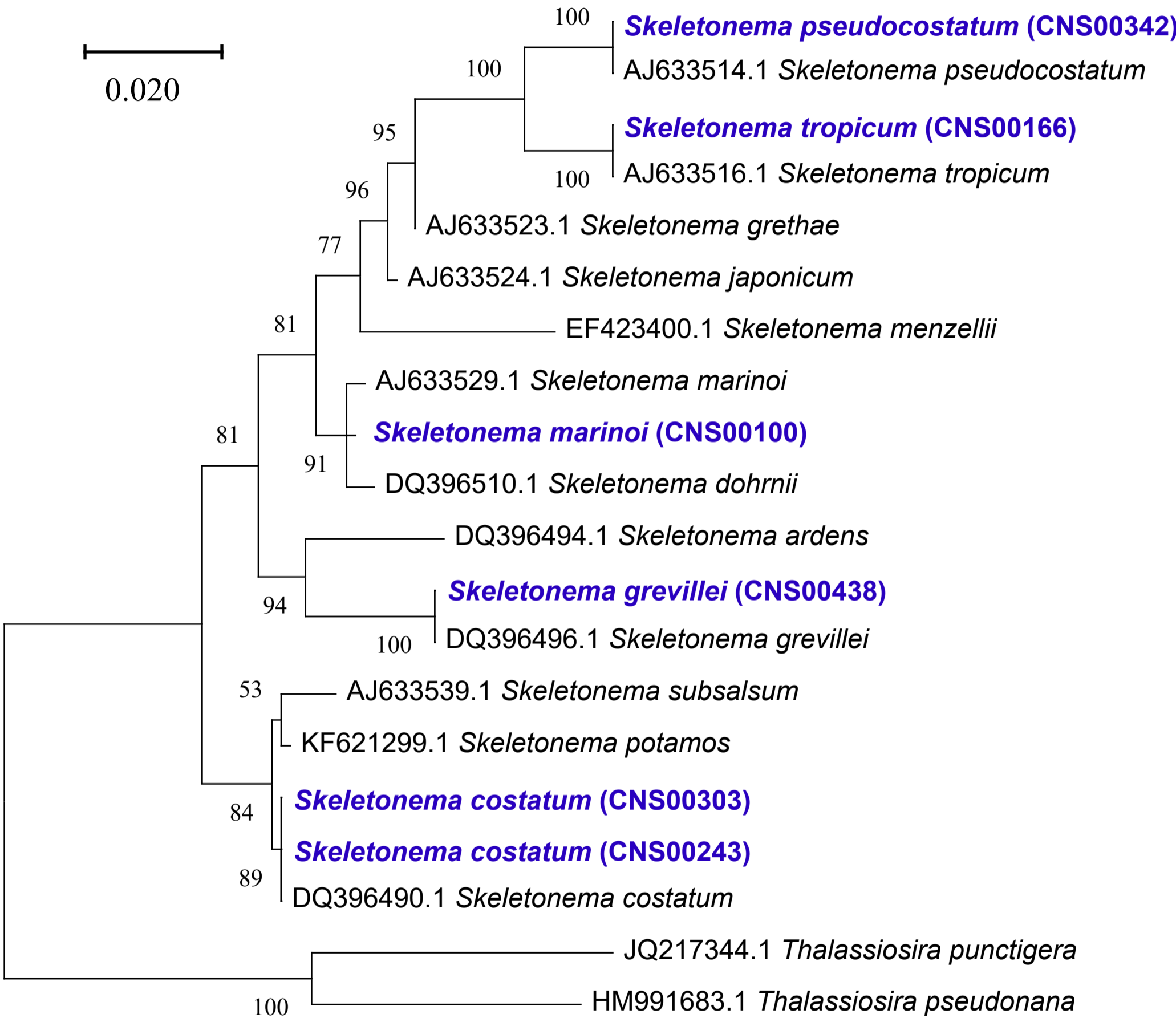

**C** *rbcL*

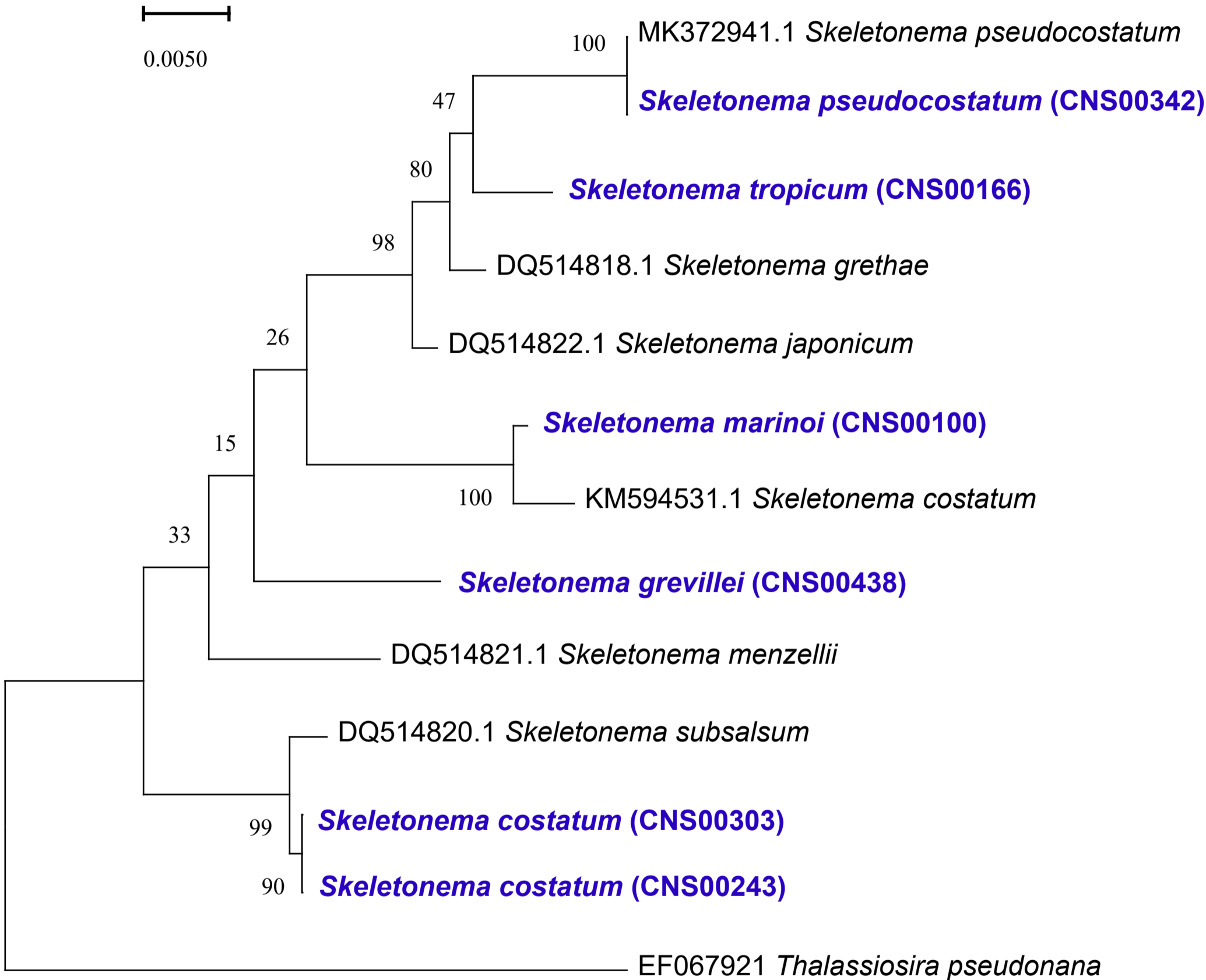

Supplement: Supplementary file 2 — Additional file 2. The phylogenetic analysis of Skeletonema species and outgroups using 18S rDNA (A), the D1-D3 region of 28S rDNA gene (B) and rbcL (C). Phylogenetic trees were generated using the Maximum Likelihood (ML) method with 1000 bootstrap replicates. Evolutionary models for 18S rDNA, the D1-D3 region of 28S rDNA gene, and rbcL were Tamura 3-parameter model with gamma distribution (G = 0.617), Kimura 2-parameter model with gamma distribution (G = 0.297), and Tamura 3-parameter with gamma distribution (G = 0.689). [file 12864_2021_7999_MOESM2_ESM.pdf]

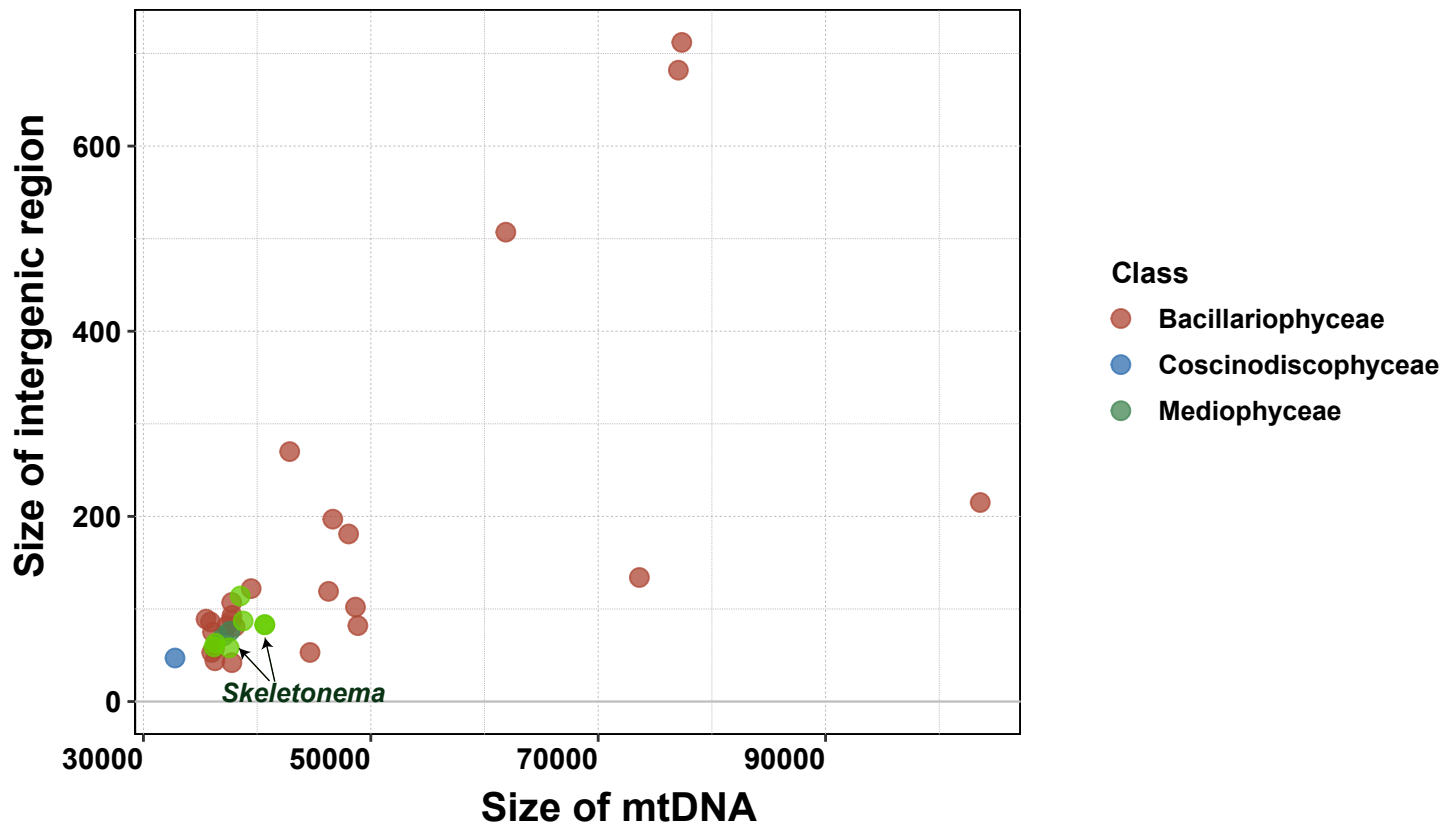

Supplement: Supplementary file 4 — Additional file 4. The intergenic region sizes and mtDNAs sizes of completed mtDNAs in the Bacillariophyta. [file 12864_2021_7999_MOESM4_ESM.pdf]

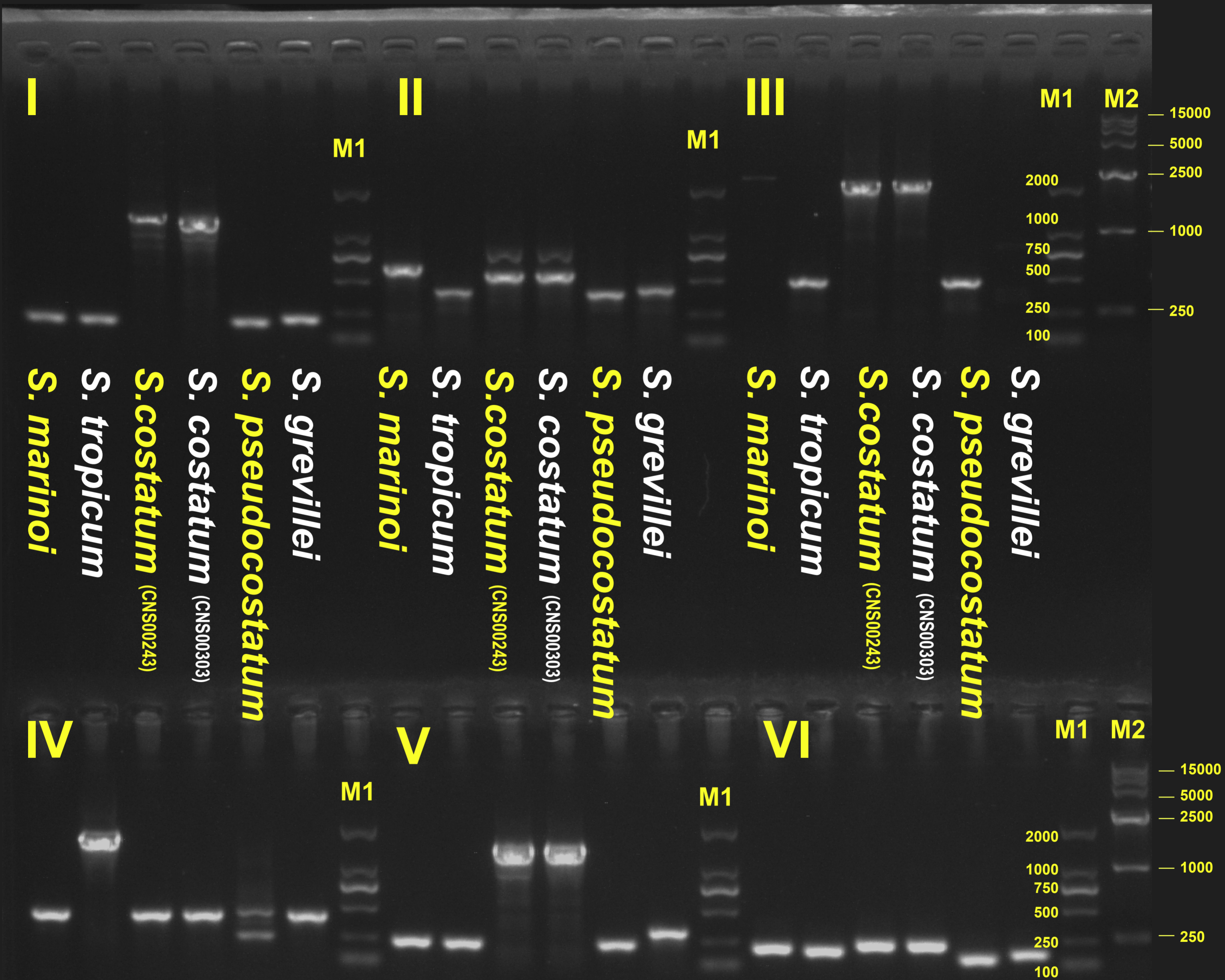

Supplement: Supplementary file 6 — Additional file 6. The agarose gels image of PCR products of seven regions among the six Skeletonema mtDNAs. The full-length gels are presented in Additional file 15. For each region (I-VI), the brands sequences were S. marinoi, S. tropicum, S. costatum (CNS00243), S. costatum (CNS00303), S. pseudocostatum and S. grevillei, respectively. The brands of Marker M1 and M2 were on the right. [file 12864_2021_7999_MOESM6_ESM.pdf]

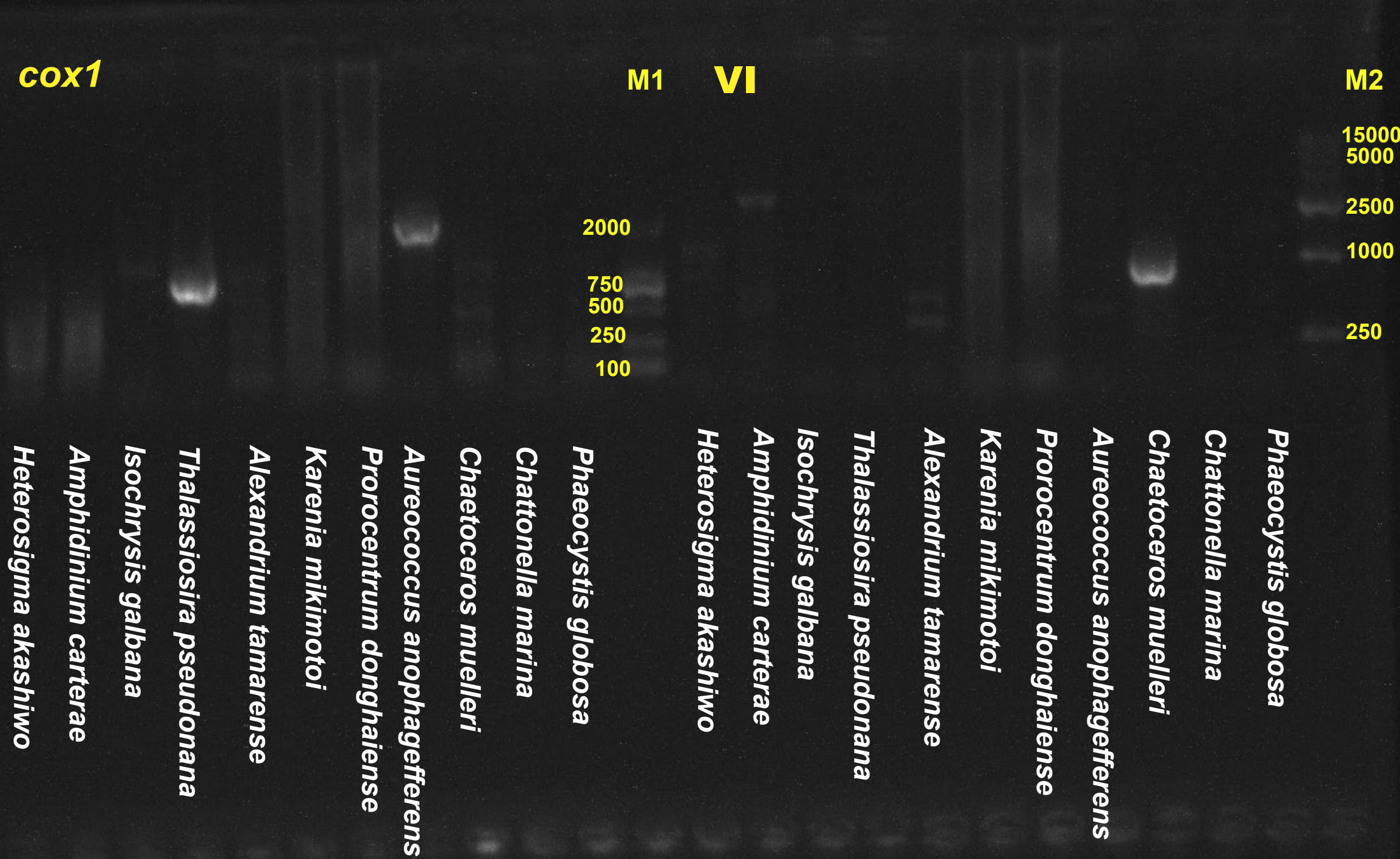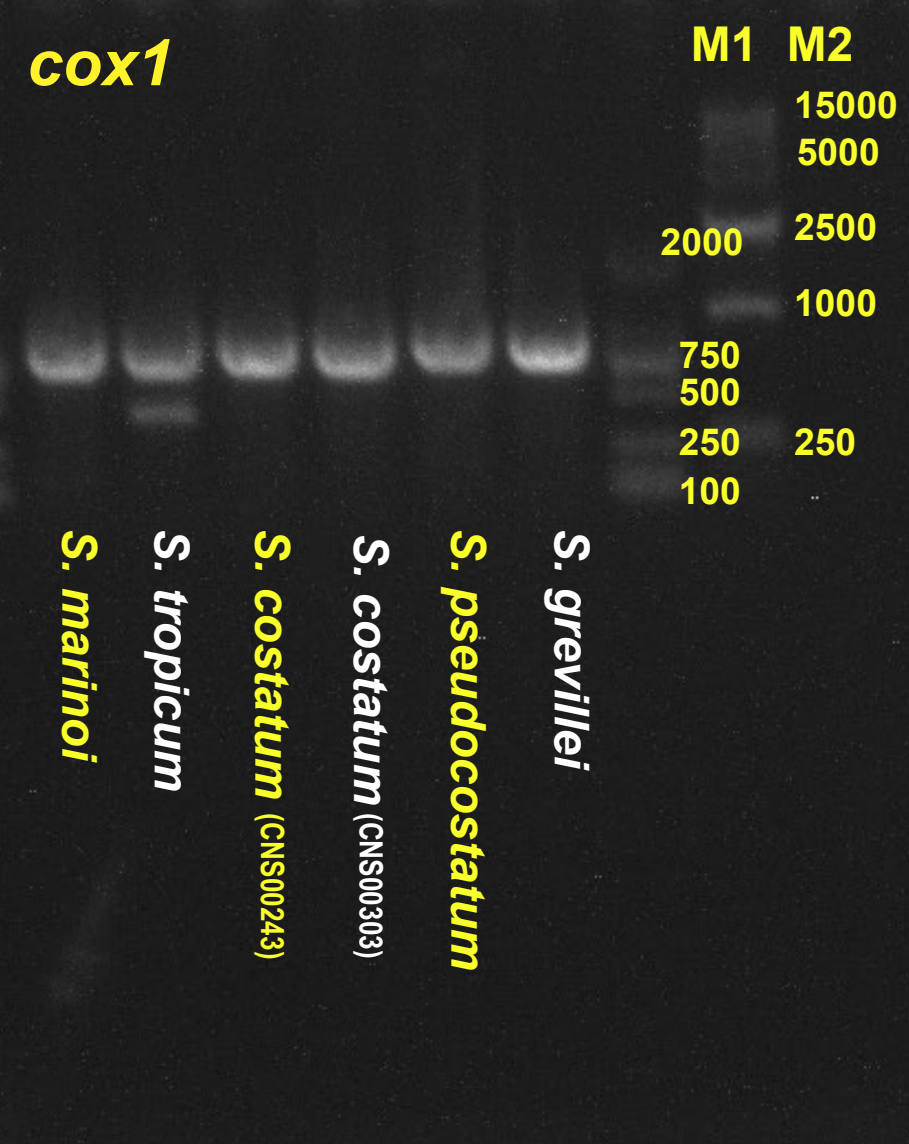

Supplement: Supplementary file 8 — Additional file 8. The agarose gels image of PCR products for cox1 and region VI among Skeletonema species and other eleven species (Thalassiosira pseudonana, Chaetoceros muelleri, Heterosigma akashiwo, Aureococcus anophagefferens, Chattonella marina, Amphidinium carterae, Alexandrium tamarense, Karenia mikimotoi, Prorocentrum donghaiense, Isochrysis galbana and Phaeocystis globosa). The primers of cox1 gene were, F: GGAACTTTATATTTAATYTTTGGWGC, R: AATACCAGAATTAGCAAGAACAAC [40]. The full-length gels are presented in Additional file 16. [file 12864_2021_7999_MOESM8_ESM.pdf]

A

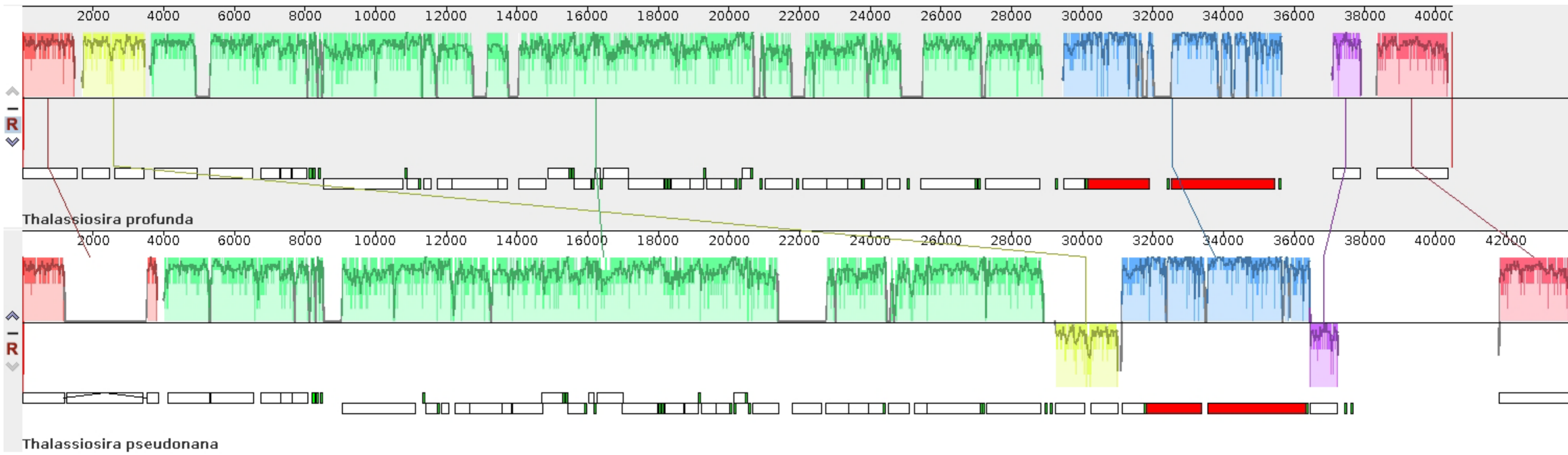

B

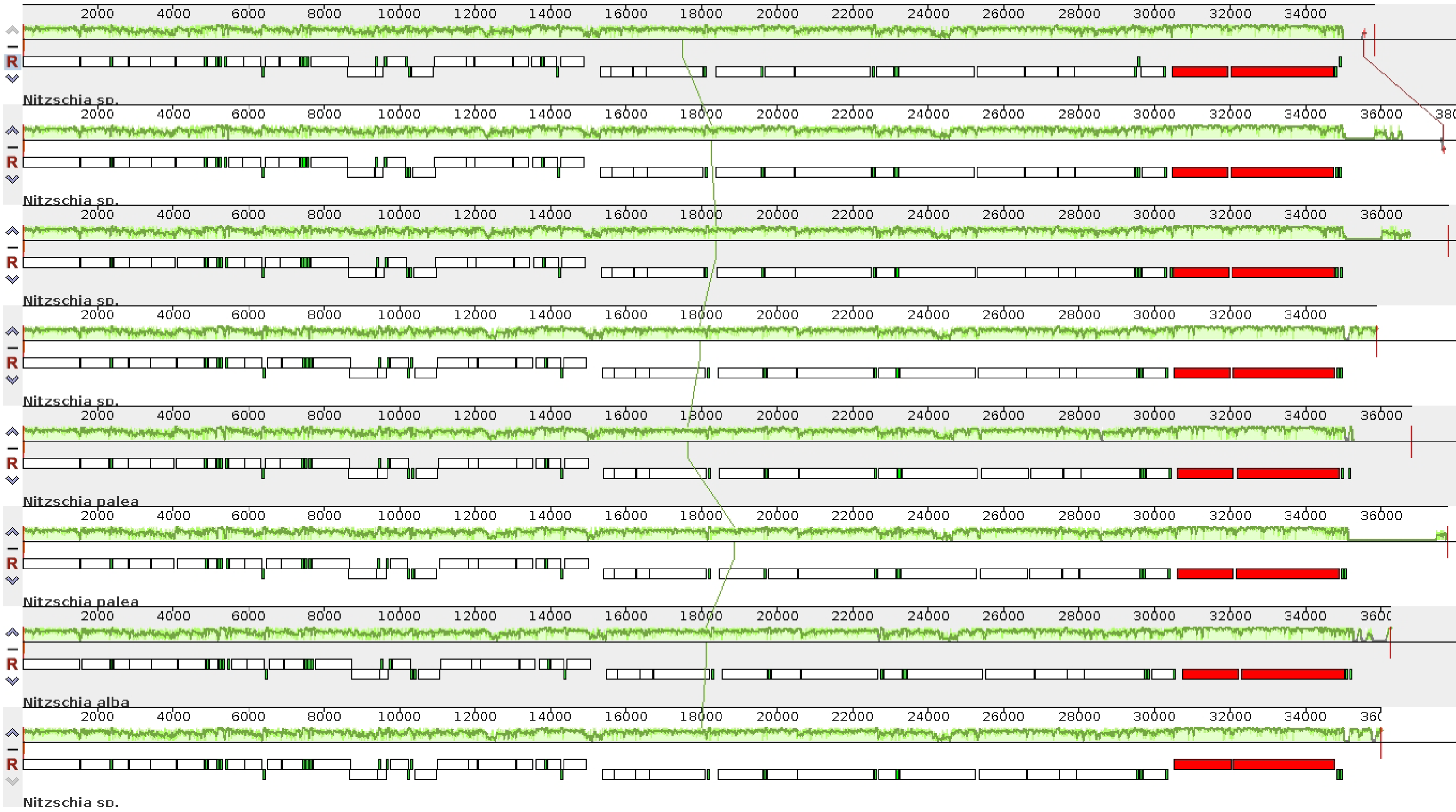

C

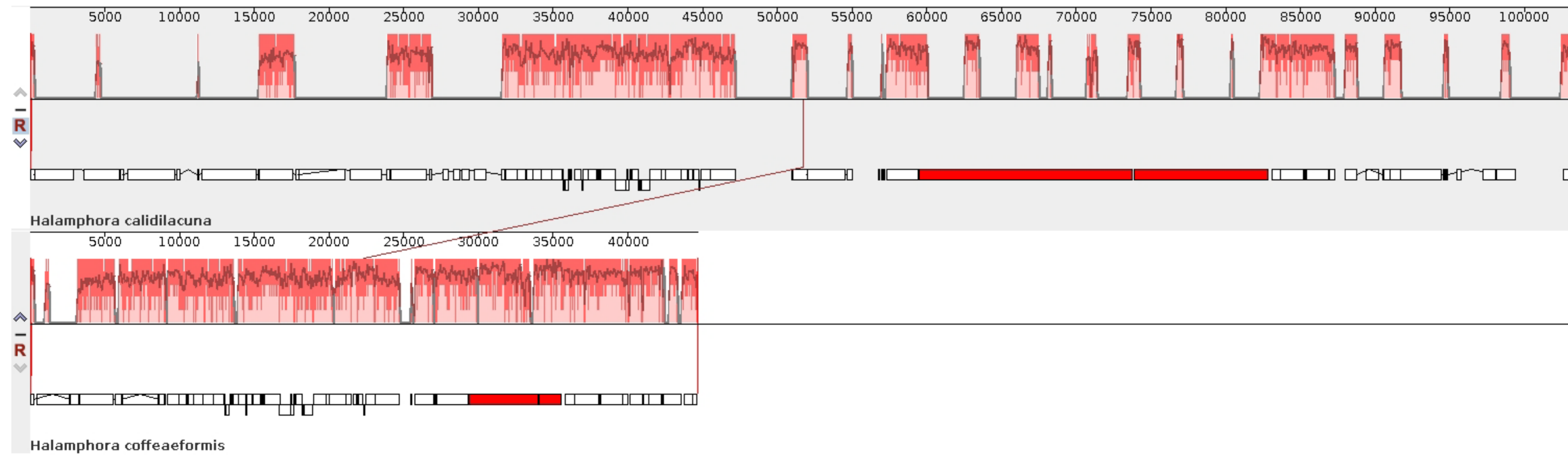

Supplement: Supplementary file 9 — Additional file 9. Synteny relationships among two Thalassiosira mtDNAs (A); eight Nitzschia mtDNAs (B); two Halamphora mtDNAs (C) based Mauve analysis. [file 12864_2021_7999_MOESM9_ESM.pdf]

A (*S. marinoi*)

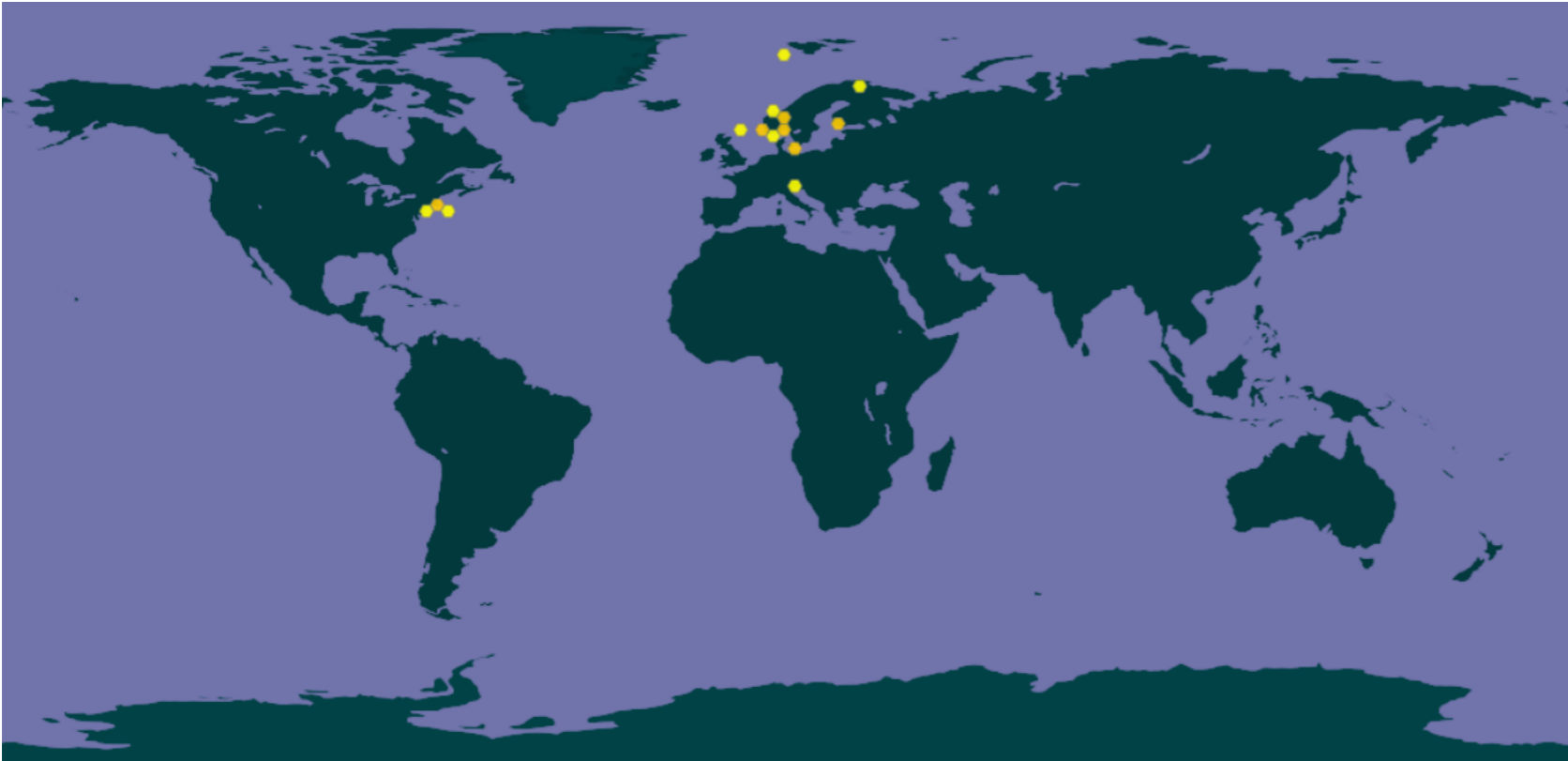

D (*S. costatum*)

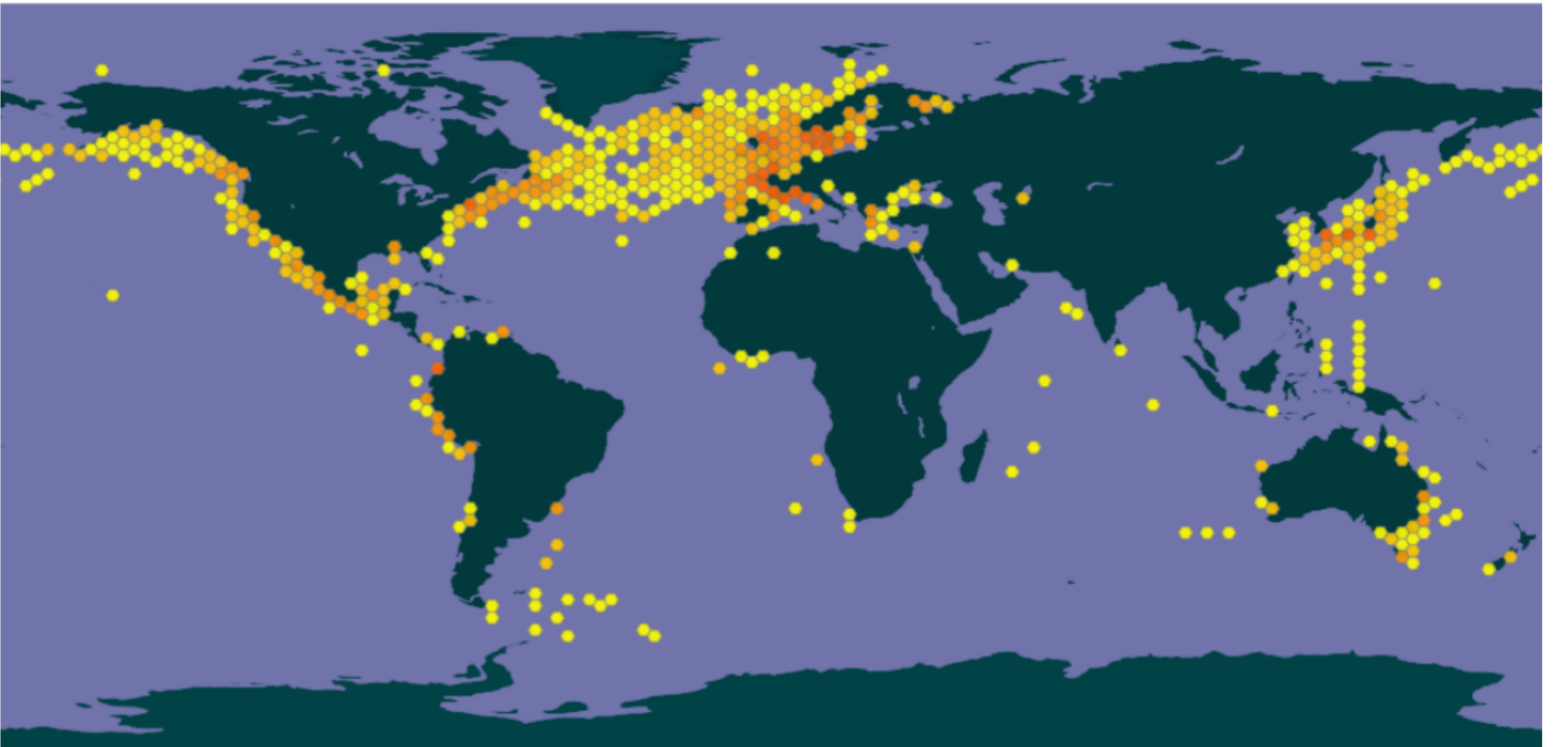

B (*S. tropicum*)

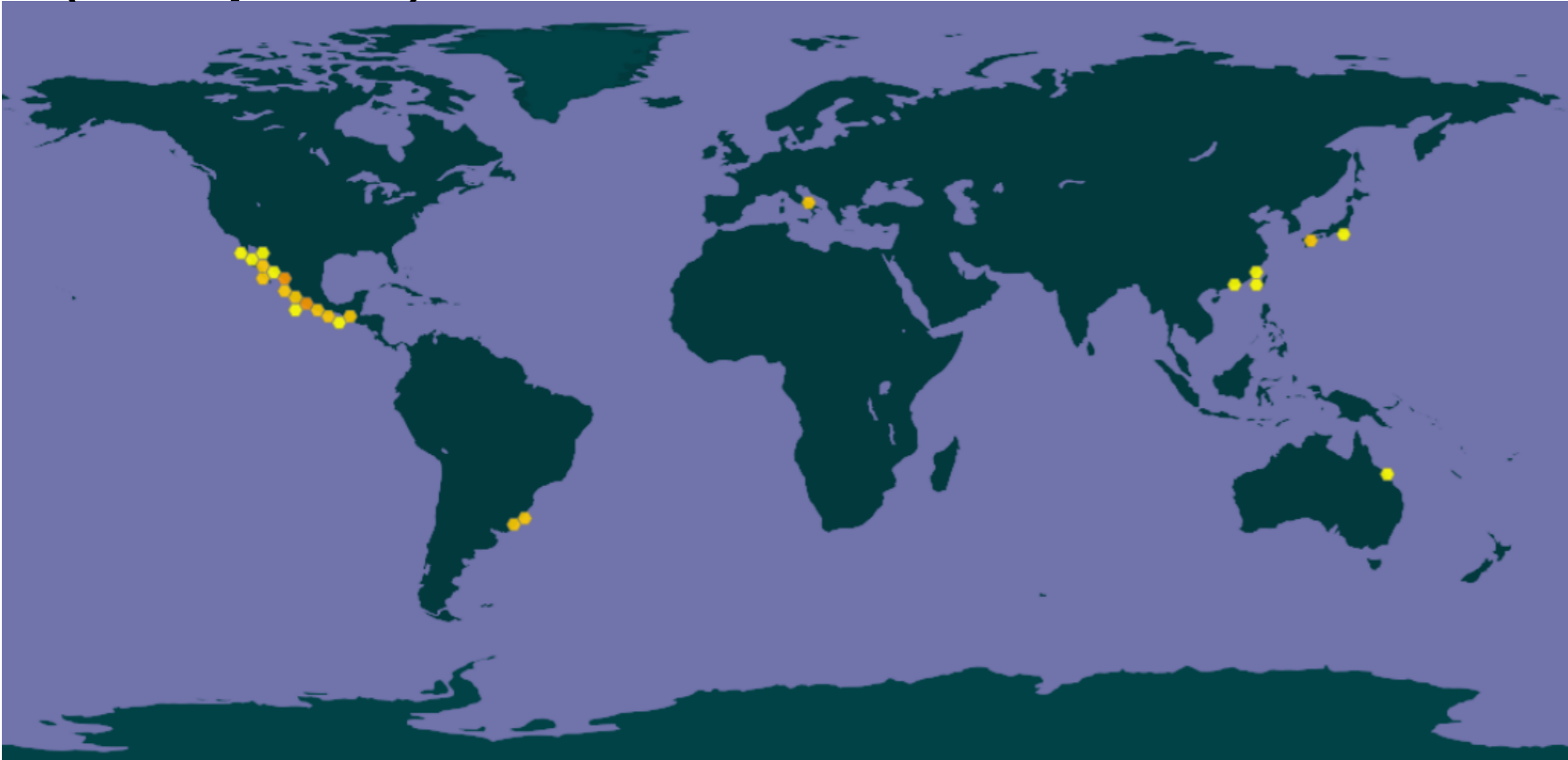

E (*S. grevillei*)

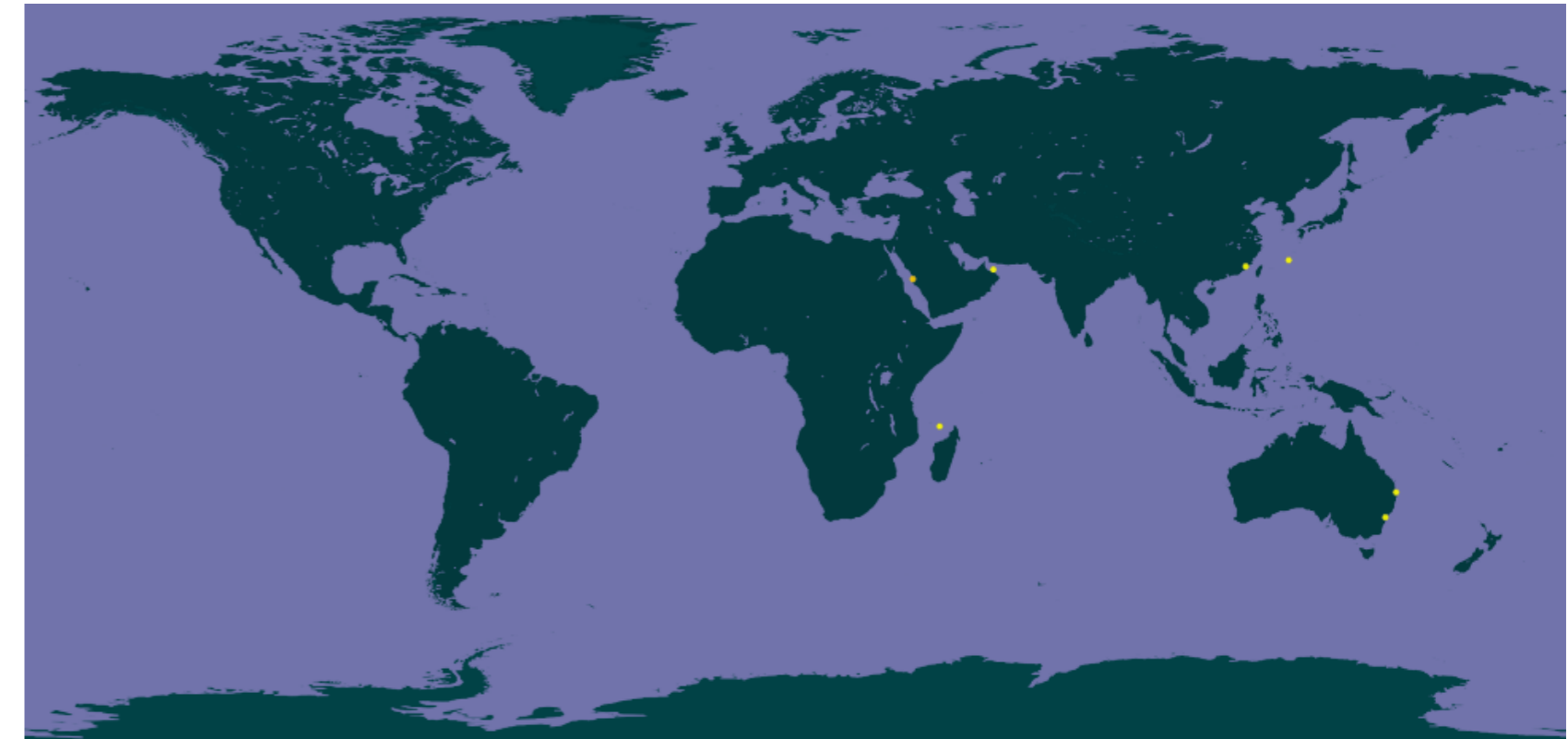

C (*S. pseudocostatum*)

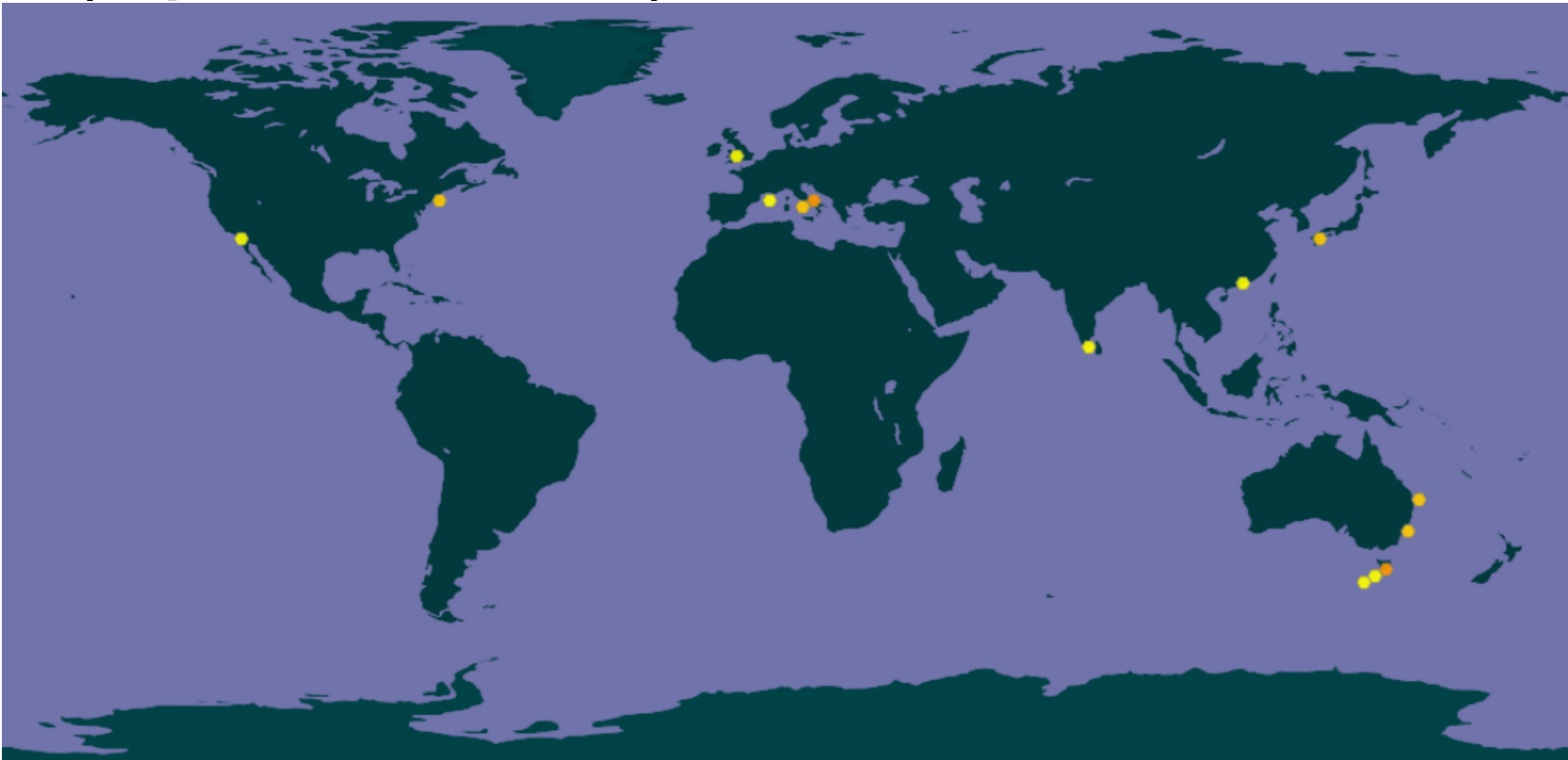

Supplement: Supplementary file 10 — Additional file 10. The distributions of five Skelentonema species from GBIF (https://www.gbif.org/). [file 12864_2021_7999_MOESM10_ESM.pdf]

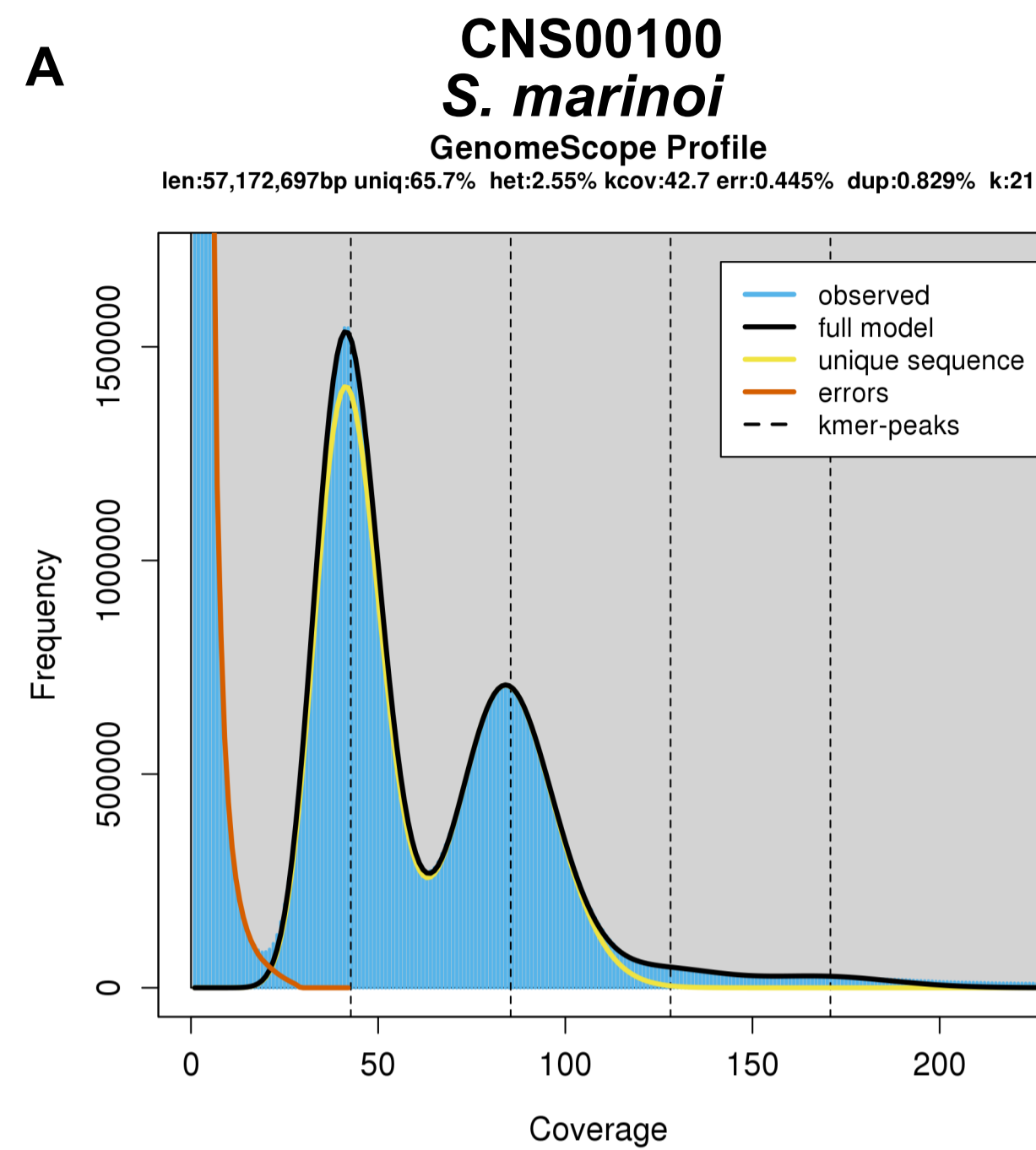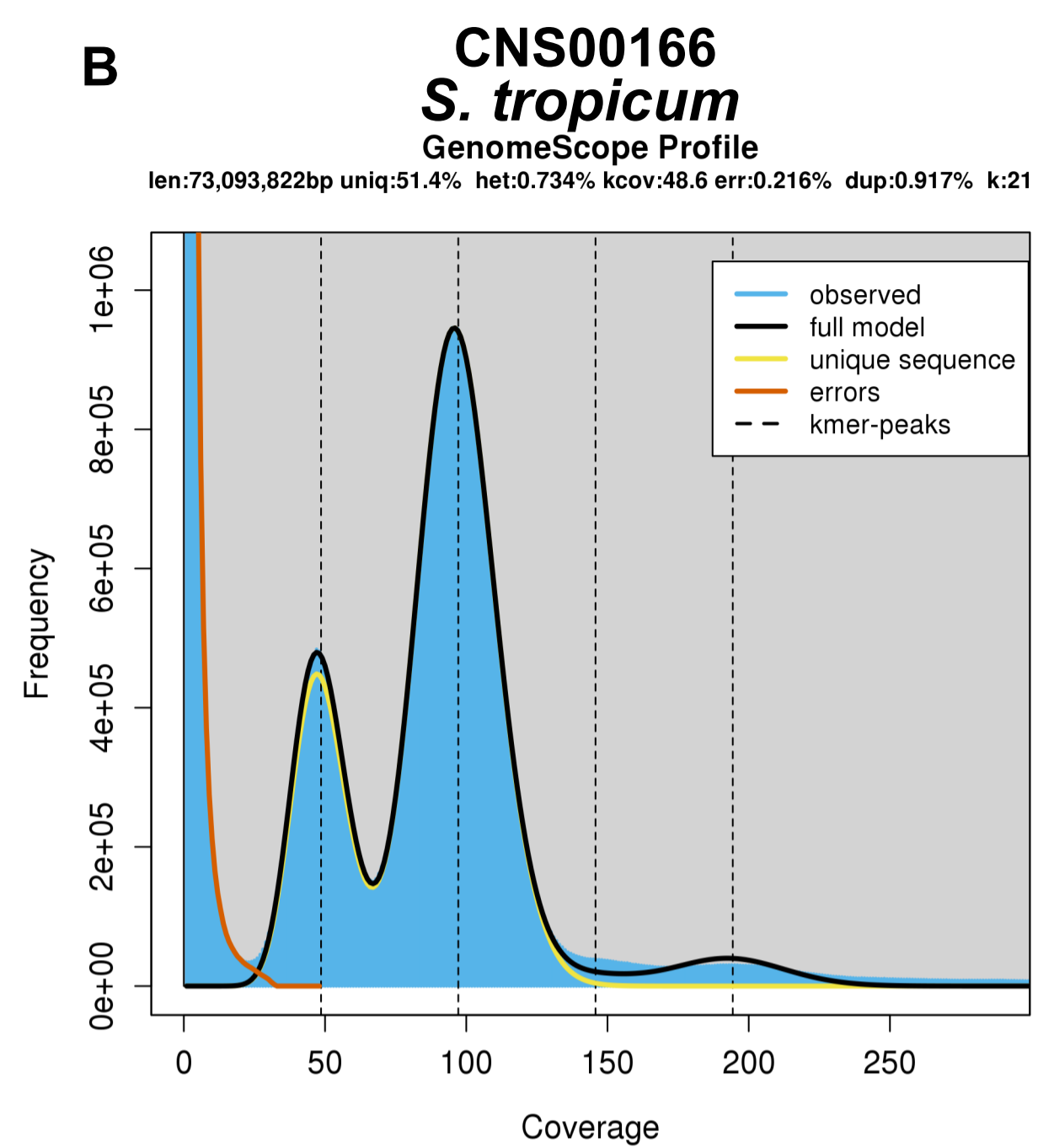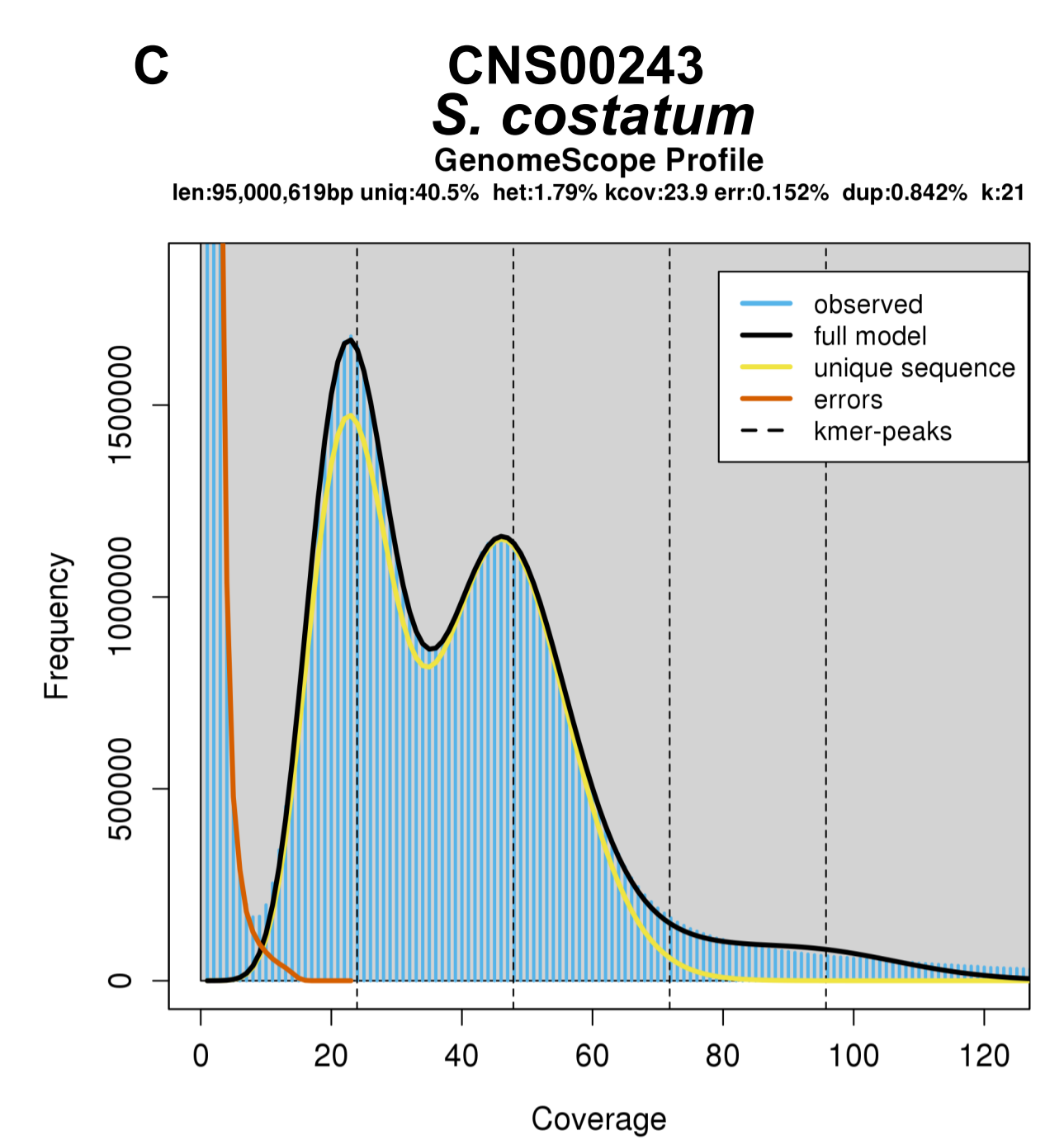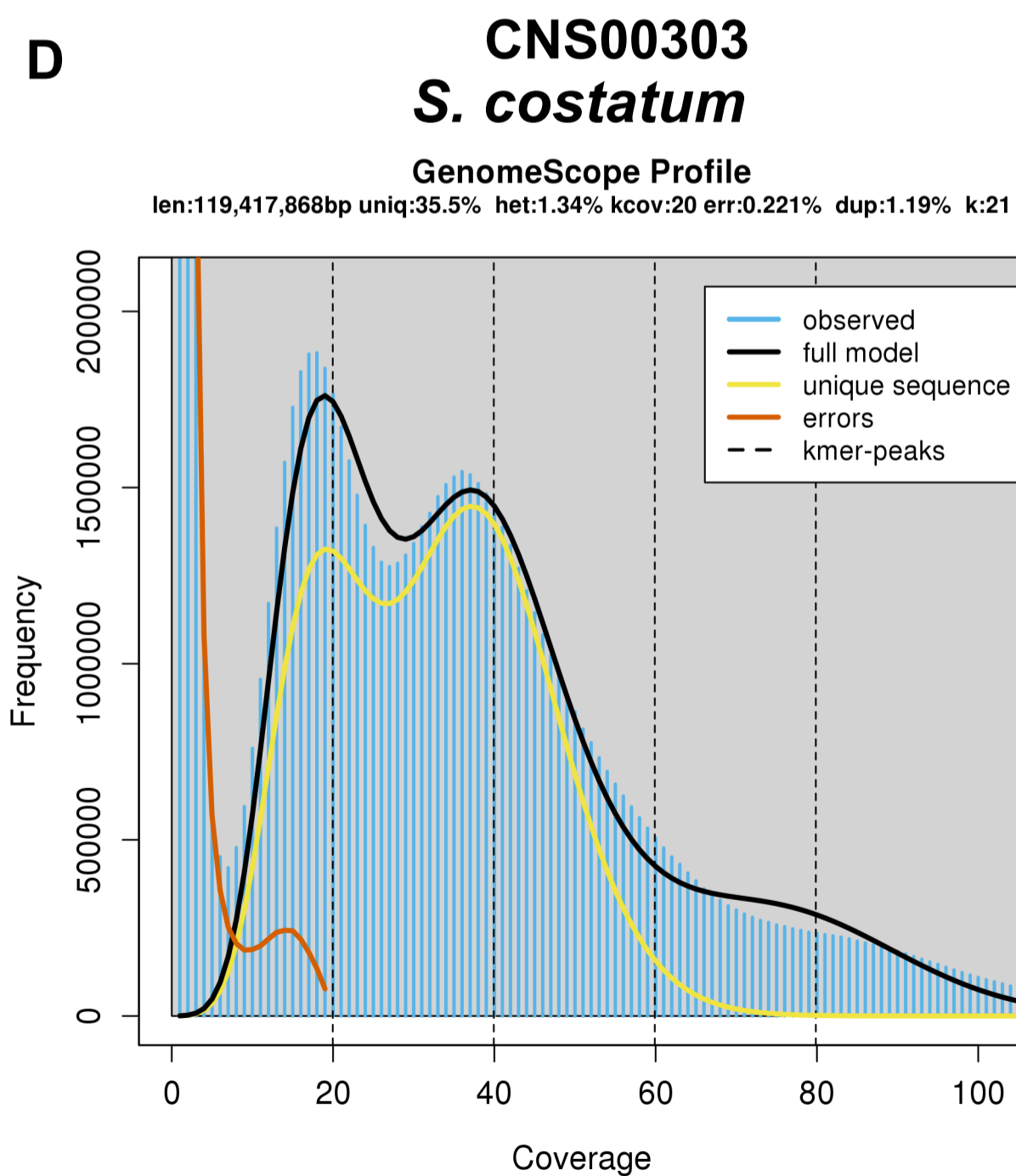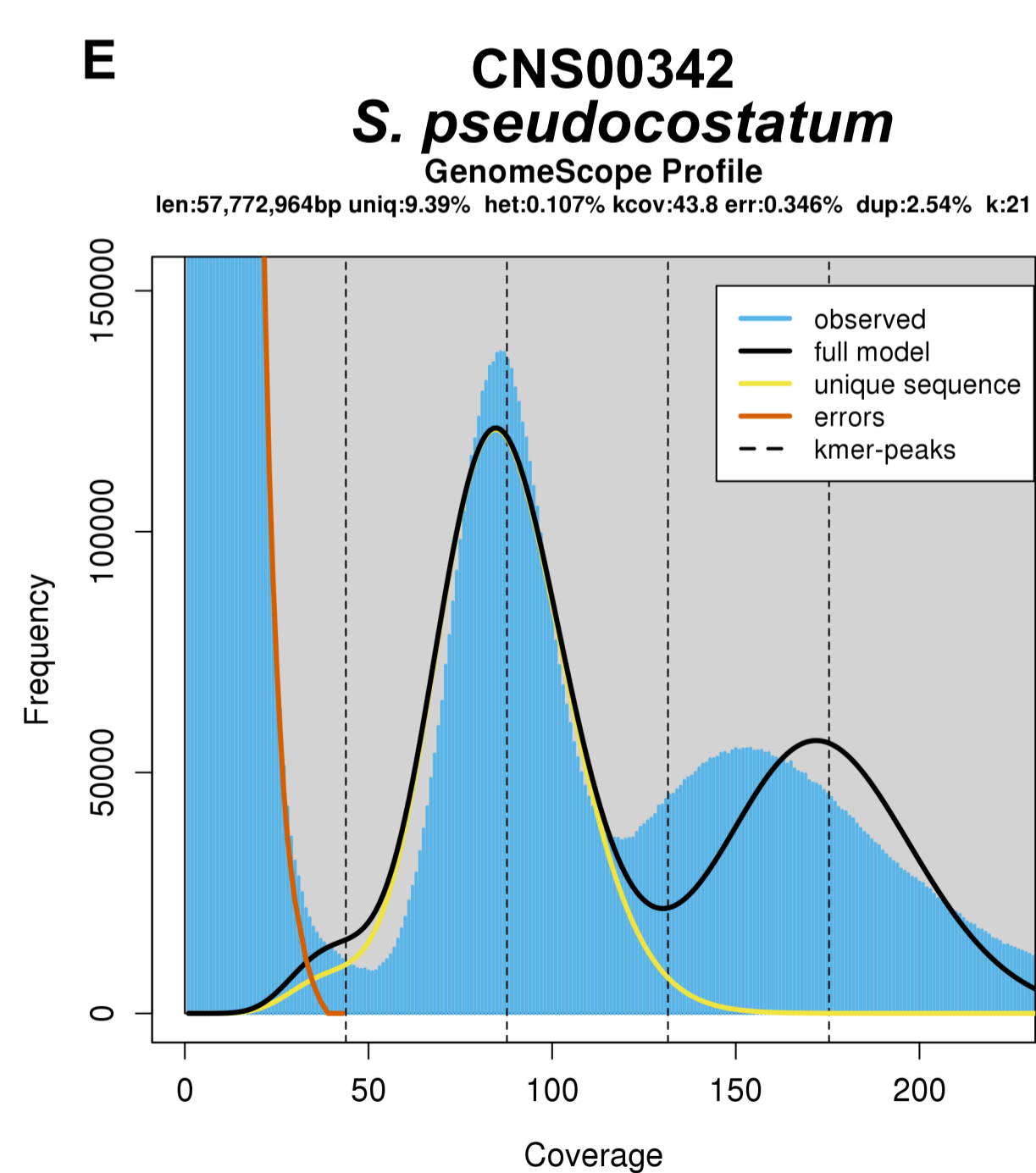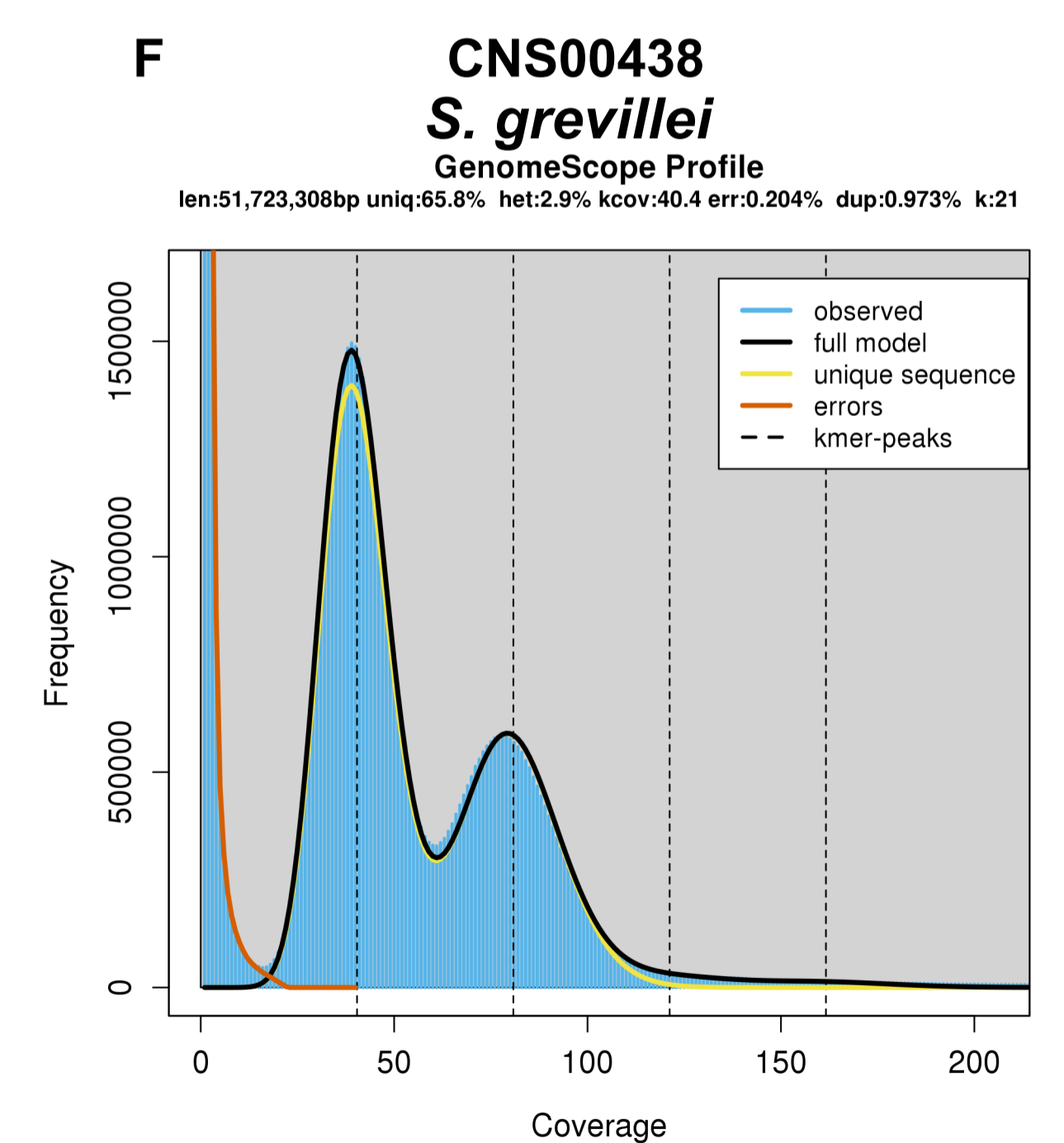

Supplement: Supplementary file 12 — Additional file 12. The genomic assessment of six Skeletonema strains. [file 12864_2021_7999_MOESM12_ESM.pdf]

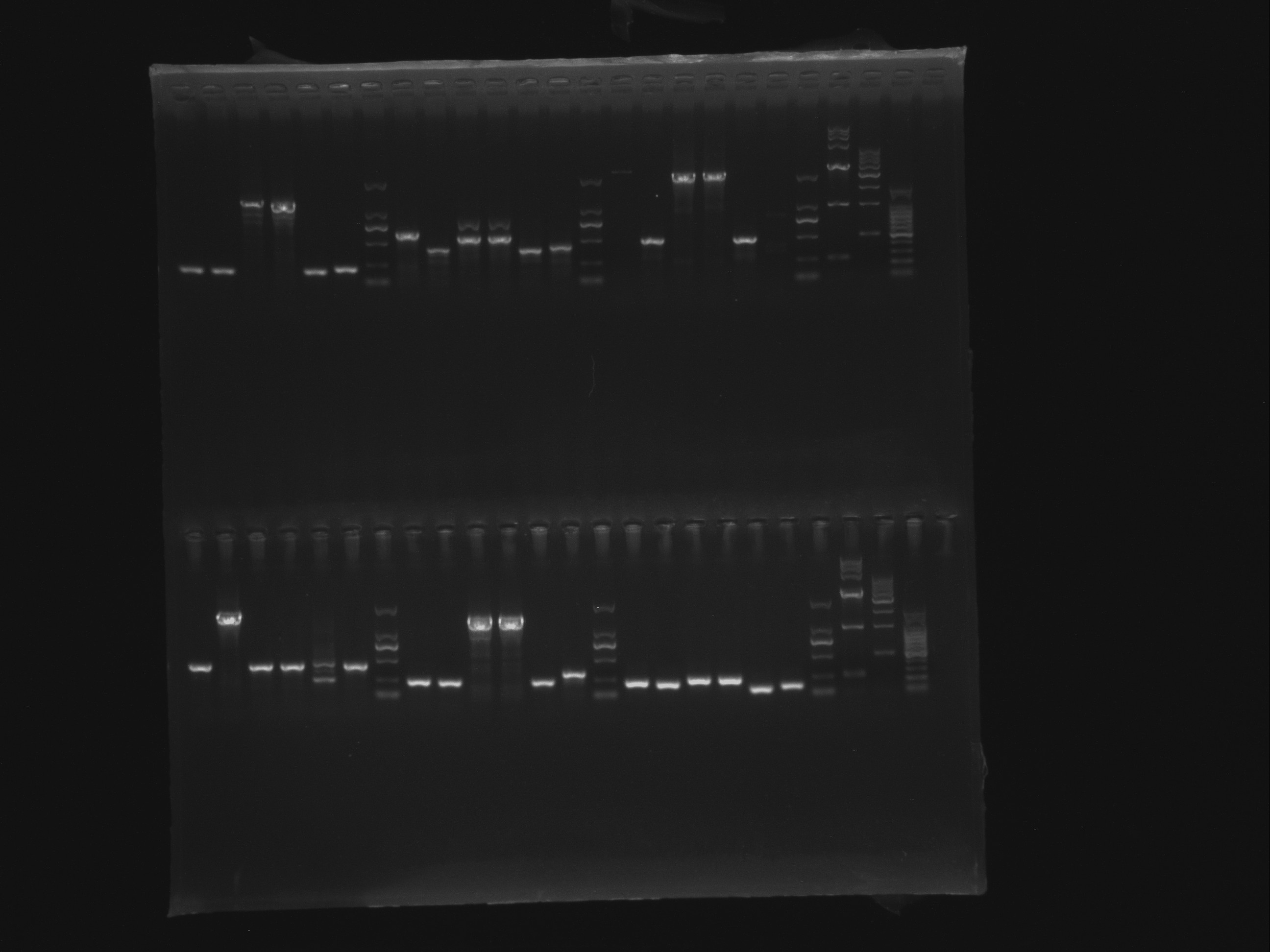

Supplement: Supplementary file 15 — Additional file 15. The full-length gels used in Fig. 5 and Additional file 7. [file 12864_2021_7999_MOESM15_ESM.jpg]
