# Supplementary material for: Comparative analysis of full-length mitochondrial genomes of five Skeletonema species reveals conserved genome organization and recent speciation
Source: BMC Genomics. 2021 Oct 15;22:746. doi: 10.1186/s12864-021-07999-z (PMC8520197; doi:10.1186/s12864-021-07999-z)
Supplement: Supplementary file 3 — Additional file 3. Genome features of 36 mtDNAs from the Phylum Bacillariophyta. [file 12864_2021_7999_MOESM3_ESM.docx]

**Additional file 3** Genome features of 36 mtDNAs from the Phylum Bacillariophyta [1, 2]

| **Class** | **Species** | **Accession number** | **Size**  **(bp)** | **A+T**  **(%)** | **Reference** |
| --- | --- | --- | --- | --- | --- |
| **Mediophyceae(5)** | *Odontella regia* | MW018491 | 37617 | 73.4 | [1] |
|  | *Lithodesmium undulatum* | MW023083 | 37057 | 75.3 | [1] |
|  | *Thalassiosira pseudonana* | NC_007405 | 43,827 | 69.9 | [3] |
|  | *Thalassiosira profunda* | MW013551 | 40,470 | 69.0 | [2] |
|  | *Skeletonema marinoi* | NC_028615 | 38,515 | 70.3 | [4] |
|  | *Toxarium undulatum* | NC_037988 | 40,429 | 69.9 | [5] |
| **Coscinodiscophyceae(1)** | *Melosira undulata* | NC_037728 | 32,777 | 78.4 | [6] |
| **Bacillariophyceae(29)** | *Psammoneis japonica* | NC_037989 | 73,622 | 69.2 | [5] |
|  | *Cylindrotheca closterium* | NC_037986 | 37,784 | 67.9 | [5] |
|  | *Fragilariopsis kerguelensis* | LR812619 | 37,348 | 68.6 | - |
|  | *Nitzschia palea* | MH297491 | 37,754 | 69.1 | [7] |
|  | *Nitzschia palea* (nearly complete) | AP018512 | >36,830 | - | [8] |
|  | *Nitzschia alba* | NC_037729 | 36,252 | 71.6 | [6] |
|  | *Nitzschia* sp. | AP018507 | 38,056 | 69.5 | [8] |
|  | *Nitzschia* sp. | AP018509 | 37,792 | 69.8 | [8] |
|  | *Nitzschia* sp. | NC_037990 | 36,012 | 71.1 | [5] |
|  | *Nitzschia* sp. | AP018510 | 35,897 | 70.8 | [8] |
|  | *Nitzschia* sp. (nearly complete) | AP018505 | >35,839 | - | [8] |
|  | *Pseudo-nitzschia multiseries* | NC_027265 | 46,283 | 68.9 | [9] |
|  | *Didymosphenia geminata* | NC_032171 | 37,765 | 73.1 | [10] |
|  | *Entomoneis* sp. | MF997419 | 36,078 | 72.2 |  |
|  | *Halamphora calidilacuna* | MF997424 | 103,605 | 68.8 | [6] |
|  | *Halamphora coffeaeformis* | NC_037727 | 44,653 | 67.1 | [6] |
|  | *Berkeleya fennica* | NC_026126 | 35,509 | 70.2 | [11] |
|  | *Fistulifera solaris* | NC_027978 | 39,476 | 71.9 | [12] |
|  | *Haslea nusantara* | NC_044492 | 36,288 | 70.8 | [13] |
|  | *Navicula ramosissima* | NC_031848 | 48,652 | 68.9 | [11] |
|  | *Phaeodactylum tricornutum* | MN956530 | 77,055 | 65.3 | - |
|  | *Phaeodactylum tricornutum* | NC_016739 | 77,356 | 65.0 | [14] |
|  | *Proschkinia* sp. | MH800316 | 48,863 | 70.4 | [15] |
|  | *Surirella* sp. | MF997423 | 42,867 | 72.6 | [6] |
|  | Endosymbiont of *Kryptoperidinium foliaceum* (partial) | JN378734 | >39,686 | - | [16] |
|  | Endosymbiont of *Durinskia baltica* (partial) | JN378735 | >35,505 | - | [16] |
|  | *Eunotia naegelii* | NC_037987 | 48,049 | 72.9 | [5] |
|  | *Asterionella formosa* | NC_032029 | 61,877 | 73.3 | [17] |
|  | *Synedra acus* (*Ulnaria acus*) | NC_013710 | 46,657 | 68.3 | [18] |

**Reference**

1. Wang Y, Chen Y, Wang J, Liu F, Chen N: **Mitochondrial genome of the harmful algal bloom species Odontella regia (Mediophyceae, Bacillariophyta)**. *Journal of Applied Phycology* 2021.

2. Liu K, Liu S, Chen Y, Liu F, Zhao Y, Chen N: **Complete mitochondrial genome of Thalassiosira profunda (Mediophyceae, Bacillariophyta)**. *Mitochondrial DNA Part B* 2021, **accept**.

3. Armbrust EV, Berges JA, Bowler C, Green BR, Martinez D, Putnam NH, Zhou SG, Allen AE, Apt KE, Bechner M *et al*: **The genome of the diatom Thalassiosira pseudonana: Ecology, evolution, and metabolism**. *Science* 2004, **306**(5693):79-86.

4. An SM, Kim SY, Noh JH, Yang EC: **Complete mitochondrial genome of Skeletonema marinoi (Mediophyceae, Bacillariophyta), a clonal chain forming diatom in the west coast of Korea**. *Mitochondrial DNA* 2017, **28**(1-2):19-20.

5. Guillory WX, Onyshchenko A, Ruck EC, Parks M, Nakov T, Wickett NJ, Alverson AJ: **Recurrent Loss, Horizontal Transfer, and the Obscure Origins of Mitochondrial Introns in Diatoms (Bacillariophyta)**. *Genome Biology and Evolution* 2018, **10**(6):1504-1515.

6. Pogoda CS, Keepers KG, Hamsher SE, Stepanek JG, Kane NC, Kociolek JP: **Comparative analysis of the mitochondrial genomes of six newly sequenced diatoms reveals group II introns in the barcoding region of cox1**. *Mitochondrial DNA Part A* 2019, **30**(1):43-51.

7. Crowell RM, Nienow JA, Cahoon AB: **The complete chloroplast and mitochondrial genomes of the diatom Nitzschia palea (Bacillariophyceae) demonstrate high sequence similarity to the endosymbiont organelles of the dinotom Durinskia baltica**. *Journal of Phycology* 2019, **55**(2):352-364.

8. Kamikawa R, Azuma T, Ishii K, Matsuno Y, Miyashita H: **Diversity of Organellar Genomes in Non-photosynthetic Diatoms**. *Protist* 2018, **169**(3):351-361.

9. Yuan X-L, Cao M, Bi G-Q: **The complete mitochondrial genome of *Pseudo-nitzschia multiseries* (Baciuariophyta)**. *Mitochondrial DNA Part A* 2016, **27**(4):2777-2778.

10. Aunins AW, Hamilton D, King TL: **The complete mitochondrial genome of the stalk-forming diatom *Didymosphenia Geminata***. *Mitochondrial DNA Part B-Resources* 2018, **3**(2):676-677.

11. An SM, Noh JH, Choi DH, Lee JH, Yang EC: **Repeat region absent in mitochondrial genome of tube-dwelling diatom *Berkeleya fennica* (Naviculales, Bacillariophyceae)**. *Mitochondrial DNA Part A* 2016, **27**(3):2137-2138.

12. Tang X, Bi G: **Complete mitochondrial genome of *Fistulifera solaris* (Bacillariophycidae)**. *Mitochondrial DNA Part A* 2016, **27**(6):4405-4406.

13. Prasetiya FS, Gastineau R, Poulin M, Lemieux C, Turmel M, Syakti AD, Hardivillier Y, Widowati I, Risjani Y, Iskandar I *et al*: ***Haslea nusantara* (Bacillariophyceae), a new blue diatom from the Java Sea, Indonesia: morphology, biometry and molecular characterization**. *Plant Ecology and Evolution* 2019, **152**(2):188-202.

14. Oudot-Le Secq MP, Green BR: **Complex repeat structures and novel features in the mitochondrial genomes of the diatoms *Phaeodactylum tricornutum* and *Thalassiosira pseudonana***. *Gene* 2011, **476**(1-2):20-26.

15. Gastineau R, Kim S-Y, Lemieux C, Turmel M, Witkowski A, Park J-G, Kim B-S, Mann DG, Theriot EC: **Complete mitochondrial genome of a rare diatom (Bacillariophyta) *Proschkinia* and its phylogenetic and taxonomic implications**. *Mitochondrial DNA Part B-Resources* 2019, **4**(1):25-26.

16. Imanian B, Pombert J-F, Dorrell RG, Burki F, Keeling PJ: **Tertiary Endosymbiosis in Two Dinotoms Has Generated Little Change in the Mitochondrial Genomes of Their Dinoflagellate Hosts and Diatom Endosymbionts**. *Plos One* 2012, **7**(8).

17. Villain A, Kojadinovic M, Puppo C, Prioretti L, Hubert P, Zhang Y, Gregori G, Roulet A, Roques C, Claverie J-M *et al*: **Complete mitochondrial genome sequence of the freshwater diatom *Asterionella formosa***. *Mitochondrial DNA Part B-Resources* 2017, **2**(1):97-98.

18. Ravin NV, Galachyants YP, Mardanov AV, Beletsky AV, Petrova DP, Sherbakova TA, Zakharova YR, Likhoshway YV, Skryabin KG, Grachev MA: **Complete sequence of the mitochondrial genome of a diatom alga Synedra acus and comparative analysis of diatom mitochondrial genomes**. *Current Genetics* 2010, **56**(3):215-223.
