# Supplementary material for: Comparative analysis of full-length mitochondrial genomes of five Skeletonema species reveals conserved genome organization and recent speciation
Source: BMC Genomics. 2021 Oct 15;22:746. doi: 10.1186/s12864-021-07999-z (PMC8520197; doi:10.1186/s12864-021-07999-z)
Supplement: Supplementary file 7 — Additional file 7. The primes for the region I to region VI among the Skeletonema genus. F in the primer names meant forward primer; R in the primer names meant reverse primer. I to VI in the primer names were corresponding to region I to region VI. [file 12864_2021_7999_MOESM7_ESM.docx]

**Additional file 7** The primes for the region I to region VI among the *Skeletonema* genus. F in the primer names means forward primer; R in the primer names means reverse primer. I to VI in the primer names were corresponding to region I to region VI.

| **Primer name** | **Primer sequence** |
| --- | --- |
| I-F | TTCAAAACCAAAGGTCAA |
| I-R | GAATAACTGTCAAAGGTAAGAT |
| II-F | TATGGTAGCAATAATGTGG |
| II-R | TAATAAAACGGAAACGAC |
| III-F | TTCCGTGGATAATCAAAT |
| III-R | GTCTATTGGTCATTTATTGTAT |
| IV-F | CGTTGAGGTATCCATTTA |
| IV-R | TTCTTTTGGTCTAGCATT |
| V-F | TTGAAACTGTTCTAAAGGAG |
| V-R | CCTGATAAGCGTAAGGTC |
| VI-F | ACTACTGACCTTACGCTTATC |
| VI-R | CGAAAAAGTTGGTGGTTCGATTCCA |
