# Supplementary material for: Comparative analysis of full-length mitochondrial genomes of five Skeletonema species reveals conserved genome organization and recent speciation
Source: BMC Genomics. 2021 Oct 15;22:746. doi: 10.1186/s12864-021-07999-z (PMC8520197; doi:10.1186/s12864-021-07999-z)
Supplement: Supplementary file 11 — Additional file 11. The sample collection and genomic assessment information of six Skeletonema strains. [file 12864_2021_7999_MOESM11_ESM.docx]

**Additional file 11:** The sample collection and genomic assessment information of six Skeletonema strains.

| **Strains** | **Sample collectionVoyage information** | **Site** | **Longitude (°E)** | **Latitude (°N)** | **Nuclear genome size(Mb)** | **Heterozygosity**  **(%)** | **repetitive rate(%)** | **Bacteria proportion**  **(%)** |
| --- | --- | --- | --- | --- | --- | --- | --- | --- |
| CNS00100 | Bohai and Yellow Sea voyage in April 2019 | B12 | 119.40 | 39.16 | 57.17 | 2.55 | 0.83 | 0.27 |
| CNS00166 | Jiaozhou Bay voyage in October 2019 | A5 | 120.52 | 36.21 | 73.09 | 0.73 | 0.92 | 0.04 |
| CNS00243 | Bohai and Yellow Sea voyage in April 2019 | H42 | 123.03 | 31.03 | 95.00 | 1.79 | 0.84 | 0.03 |
| CNS00303 | Changjiang Estuary voyage in July 2019 | A8-2 | 122.86 | 30.36 | 119.42 | 1.34 | 1.19 | 18.58 |
| CNS00342 | Beibu Gulf voyage in March 2019 | ZN5-4 | 109.33 | 21.37 | 57.77 | 0.11 | 2.54 | 20.64 |
| CNS00438 | South China Sea voyage in June 2020 | S52 | 112.00 | 18.00 | 51.72 | 2.90 | 0.97 | 0.20 |

*The Genbank ID of the full length of 18 rDNA gene; PID means the percentage identity.
