# Supplementary material for: Comparative analysis of full-length mitochondrial genomes of five Skeletonema species reveals conserved genome organization and recent speciation
Source: BMC Genomics. 2021 Oct 15;22:746. doi: 10.1186/s12864-021-07999-z (PMC8520197; doi:10.1186/s12864-021-07999-z)
Supplement: Supplementary file 13 — Additional file 13. The best-fit evolutionary model and partitioning scheme for phylogenetic analysis and molecular dating. [file 12864_2021_7999_MOESM13_ESM.docx]

**Additional file 13:** The best-fit evolutionary model and partitioning scheme for phylogenetic analysis and molecular dating. (Docx 16kb)

| Subset | Best Model | sites | Partition names |
| --- | --- | --- | --- |
| 1 | GTR+I+G | 1158 | *nad4L, atp6* |
| 2 | GTR+I+G | 1191 | *atp8, rps11* |
| 3 | GTR+G | 228 | *atp9* |
| 4 | GTR+I+G | 1551 | *cox1* |
| 5 | GTR+I+G | 3246 | *cytb, cox3, cox2* |
| 6 | GTR+I+G | 2487 | *nad11* |
| 7 | GTR+I+G | 1053 | *nad1* |
| 8 | GTR+I+G | 2895 | *nad6, nad2* |
| 9 | GTR+I+G | 543 | *nad3* |
| 10 | GTR+I+G | 3624 | *nad4, nad5* |
| 11 | GTR+I+G | 1200 | *nad7* |
| 12 | GTR+I+G | 633 | *nad9* |
| 13 | GTR+G | 1026 | *rpl14, rps8, tatA* |
| 14 | HKY+G | 429 | *rpl16* |
| 15 | GTR+I+G | 909 | *rpl2* |
| 16 | GTR+I+G | 1935 | *rps3, rpl5* |
| 17 | GTR+I+G | 1020 | *rps13, rpl6* |
| 18 | GTR+I+G | 675 | *rps10* |
| 19 | GTR+I+G | 396 | *rps12* |
| 20 | GTR+I+G | 1173 | *rps14, rps4* |
| 21 | GTR+I+G | 1011 | *rps19, rps7* |
| 22 | GTR+I+G | 789 | *tatC* |
