# Supplementary material for: Comparative analysis of full-length mitochondrial genomes of five Skeletonema species reveals conserved genome organization and recent speciation
Source: BMC Genomics. 2021 Oct 15;22:746. doi: 10.1186/s12864-021-07999-z (PMC8520197; doi:10.1186/s12864-021-07999-z)
Supplement: Supplementary file 14 — Additional file 14. Calibration points used in the divergence time analysis by PAML. [file 12864_2021_7999_MOESM14_ESM.docx]

**Additional file 14:** Calibration points used in the divergence time analysis by PAML. (Docx 21 kb)

| **Bracket 1** | **Bracket 2** | **Divergence time (Ma)** | **reference** |
| --- | --- | --- | --- |
| *Synedra* | *Fragilaria* | 56-126 | [1] |
| *Thalassiosira profunda* | *Thalassiosira pseudonana* | 30-70 | [2-4] |

Reference

1. Nakov T, Beaulieu JM, Alverson AJ: **Accelerated diversification is related to life history and locomotion in a hyperdiverse lineage of microbial eukaryotes (Diatoms, Bacillariophyta)**. *New Phytol* 2018, **219**(1):462-473.

2. Whittaker KA, Rignanese DR, Olson RJ, Rynearson TA: **Molecular subdivision of the marine diatom Thalassiosira rotula in relation to geographic distribution, genome size, and physiology**. *BMC Evol Biol* 2012, **12**:209.

3. Sorhannus U: **A nuclear-encoded small-subunit ribosomal RNA timescale for diatom evolution**. *Marine Micropaleontology* 2007, **65**(1-2):1-12.

4. Alverson AJ: **Timing marine-freshwater transitions in the diatom order Thalassiosirales**. *Paleobiology* 2014, **40**(1):91-101.
