# Supplementary material for: Comparative analysis of full-length mitochondrial genomes of five Skeletonema species reveals conserved genome organization and recent speciation
Source: BMC Genomics. 2021 Oct 15;22:746. doi: 10.1186/s12864-021-07999-z (PMC8520197; doi:10.1186/s12864-021-07999-z)
Supplement: Supplementary file 5 — Additional file 5. The alignment details of seven regions with great difference of six Skeletonema mtDNAs. [file 12864_2021_7999_MOESM5_ESM.pdf]

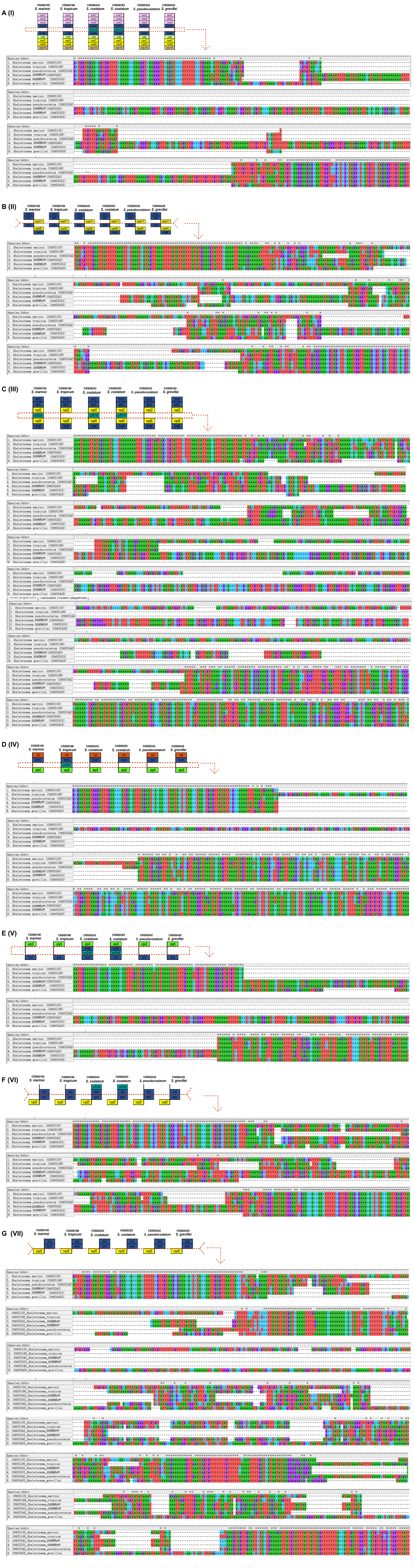
A (I)
B (II)
C (III)
D (IV)
E (V)
F (VI)
G (VII)
CNS00100 S. marinoi
CNS00166 S. tropicum
CNS00243 S. costatum
CNS00303 S. costatum
CNS00342 S. pseudocostatum
CNS00438 S. grevillei
